# Epigenetic Effects of Addictive Drugs in the Nucleus Accumbens

**DOI:** 10.3389/fnmol.2022.828055

**Published:** 2022-06-23

**Authors:** Ethan M. Anderson, Makoto Taniguchi

**Affiliations:** Department of Neuroscience, Medical University of South Carolina, Charleston, SC, United States

**Keywords:** epigenetic, substance use disorder, histone (de)acetylation, histone methlyation, nucleus accumbens, alcohol use disorder (AUD)

## Abstract

Substance use induces long-lasting behavioral changes and drug craving. Increasing evidence suggests that epigenetic gene regulation contributes to the development and expression of these long-lasting behavioral alterations. Here we systematically review extensive evidence from rodent models of drug-induced changes in epigenetic regulation and epigenetic regulator proteins. We focus on histone acetylation and histone methylation in a brain region important for drug-related behaviors: the nucleus accumbens. We also discuss how experimentally altering these epigenetic regulators via systemically administered compounds or nucleus accumbens-specific manipulations demonstrate the importance of these proteins in the behavioral effects of drugs and suggest potential therapeutic value to treat people with substance use disorder. Finally, we discuss limitations and future directions for the field of epigenetic studies in the behavioral effects of addictive drugs and suggest how to use these insights to develop efficacious treatments.

## Introduction

Substance use disorder (SUD) is defined by the DSM-5 as problematic patterns of “using alcohol or another substance that results in impairment in daily life or noticeable distress” (American Psychiatric Association, 2013). SUD contributes to major health problems in society, like the current opioid crisis in the United States (Seth et al., 2018; Volkow and Blanco, 2021). People suffering from SUD can severely impact their own personal health and negatively impact society around them, but we still only have a limited understanding of how a SUD is formed and maintained in the brain. Of particular note, our knowledge of how substance use-promoting mechanisms in the brain are maintained for years or even decades after the last use of a substance is incomplete. One possible mechanism for these long-lasting changes in the brain that promote SUD involves epigenetic changes. Epigenetic mechanisms provide a molecular basis for long-term gene regulation following interactions with the environment like using addictive substances repeatedly over time. Understanding these mechanisms is a major goal of epigenetic research on SUD. Below we will discuss how epigenetic regulation occurs, some of the evidence for epigenetic regulation in SUD in humans and in rodent models, and some challenges facing the field going forward.

### Introduction to Drug-Related Behaviors

Most of the references below discuss findings from rodent models of SUD. Broadly speaking, rodent models can be separated into two classes.

Experimenter administered (non-contingent) models. These include conditioned place preference (CPP), locomotor sensitization, and alcohol vapor exposure where the rodents have no choice in drug exposure.

Self-administration (contingent) models. These models allow the rodents more choice over when to take drugs. These assays include alcohol drinking (2-bottle choice, drinking-in-the-dark) and drug self-administration (heroin, cocaine, methamphetamine, nicotine, etc.).

As we will detail below, these contingent and non-contingent experimental models sometimes indicate a similar role of epigenetic regulators in the development or maintenance of drug reward and/or conditioned behaviors. However, in other cases, similar manipulations produce different effects depending on the behavior. In this text, we refer to one or more of these behaviors (regardless of contingent or non-contingent) as “drug-related” behaviors.

### Genetic Versus Epigenetic Mechanisms of Substance Use Disorder

Drug-related behaviors can be influenced by both genetic and epigenetic mechanisms.

#### Genetic Mechanisms of Substance Use Disorder

Genetic mechanisms involve inheritable DNA base pair differences, and a consensus of the field is that about 50% of the vulnerability to develop a SUD is genetic (Wang et al., 2012; Reilly et al., 2017). For instance, clear genetic effects exist for certain alleles of alcohol dehydrogenase (ALD) to reduce excessive alcohol use (Wang et al., 2012), and adoption studies have shown that genetic inheritance plays a stronger role than an individual’s familial environment in predicting who will develop alcohol use disorders (Schuckit et al., 1972; Goodwin et al., 1973, 1974, 1977; Reilly et al., 2017).

#### Epigenetic Mechanisms of Substance Use Disorder

In contrast to genetics, epigenetics in its simplest definition means “above” or “on top of” (“epi” – Greek) genetics and broadly refers to the ability to induce long-lasting changes based on environmental influences instead of DNA base pair differences. The term “epigenetics” has many definitions though (Deans and Maggert, 2015; Allis and Jenuwein, 2016), and here we will discuss the two most common in the literature. The first definition refers strictly to transgenerational, inherited changes depending on the environment of the offspring’s parents. The second definition involves the regulation of gene expression through changes in DNA methylation, histone post-translational modifications (PTMs), and chromatin structure. We discuss evidence for each of these below.

##### Transgenerational Epigenetic Mechanisms

Epigenetic mechanisms can influence drug-related behaviors of offspring. For instance, if rats self-administer cocaine before they reproduce, their male offspring will - surprisingly - have reductions in cocaine self-administration behavior (Vassoler et al., 2013) and cocaine locomotor sensitization in the 1st generation (F1), but not the 2nd (F2) generation (Wimmer et al., 2019). A similar finding was reported for morphine exposed fathers and their F1 and F2 offspring (Vassoler et al., 2017). In addition, nicotine exposed males sire F1 generation offspring with increased spontaneous locomotor activity and learning deficits. Furthermore, males in the F2 generation also display deficits in learning (McCarthy et al., 2018). In addition, similar findings have been shown in alcohol models, where males that had chronic alcohol exposure sire F1 offspring that later display reductions in alcohol self-administration (Nieto et al., 2022). Somewhat in contrast to these studies, however, other studies have suggested that the offspring of rats exposed to cocaine have increases in the motivation for cocaine in the F1 and F2 generations (Le et al., 2017). Together, this indicates that while most reports do show transgenerational effects, their results are not always similar. Despite the evidence for transgenerational epigenetic effects, most studies of epigenetic mechanisms in rodent models focus on a different aspect of substance-induced epigenetic regulation.

##### Environmental Epigenetic Mechanisms

A second definition of epigenetics is the regulation of gene expression caused by environmental changes. This definition is similar to our previous review (Anderson et al., 2018b) and other reviews in the field (Jaenisch and Bird, 2003; Nestler, 2013; Kenny, 2014; Allis and Jenuwein, 2016; Werner et al., 2021). These gene expression changes can occur through alterations in DNA methylation, histone post-translational modifications (PTMs), and chromatin structure as detailed below.

*DNA Methylation*. DNA can be methylated on cytosine residues when they are immediately followed by a guanine residue (CpG sites). These methylation marks can inhibit or promote transcription depending on their location on DNA (Christman et al., 1977; Bird and Southern, 1978; Desrosiers et al., 1979; Jones and Taylor, 1980) by reducing the binding of transcription initiators or by recruiting repressor proteins like methyl-CpG binding protein 2 (MeCp2) (Meehan et al., 1989, 1992; Lewis et al., 1992; Deng et al., 2010, 2014).

*Histone Post-translational Modifications*. DNA in the nucleus is wrapped around sets of 8 proteins called histones to form a nucleosome, the basic structural unit of the chromosome. There are several types of histones including H2A, H2B, H3, and H4 (Luger et al., 1997) and they can undergo a variety of PTMs on their N-terminal tails that can influence transcription and form the basis of the “histone code” (Allis and Jenuwein, 2016).

##### Histone Acetylation

The first such regulation discovered was that increased acetylation of histones results in increased transcription (Allfrey et al., 1964). This effect may be caused by increased physical accessibility for transcriptional machinery due to an electrostatic repulsion of the negatively charged phosphates of DNA by negatively charged acetyl groups on histones (Sterner and Berger, 2000; Eberharter and Becker, 2002). In addition, acetylation also recruits regulatory factors like the bromodomain-containing protein Creb-binding protein (CBP) that has histone acetyltransferase activity (HAT) and can increase transcription (Hong et al., 1993; Grunstein, 1997; Yang, 2004; Bannister and Kouzarides, 2011).

##### Histone Methylation

Histone methylation is another common histone PTM, where a methyl group is attached to a lysine or arginine (Di Lorenzo and Bedford, 2011; Benevento et al., 2015; Zhang et al., 2015). Like acetylation however, methylation also recruits regulatory factors like heterochromatin protein 1 (HP1) to alter transcription (Lachner et al., 2001; Bannister and Kouzarides, 2011; Benevento et al., 2015).

##### Other Post-translational Modifications

Many more PTMs exist like phosphorylation, sumoylation, ubiquitination, and ADP-ribosylation; however, we will not discuss these in this review.

*Chromatin Structure*. Histone- and DNA-containing nucleosomes are grouped together into chromatin. Chromatin can consist of dense, compact regions that are transcriptionally repressed and not actively transcribed known as heterochromatin. Heterochromatin can also be subcategorized into constitutive (condensed/not transcribed) and facultative (loose/transcribable under certain conditions). Chromatin can also exist in forms that are easily transcribed known as euchromatin (Huisinga et al., 2006; Delcuve et al., 2009). Drug exposure has been shown to alter chromatin accessibility status through epigenetic mechanisms through DNA methylation and histone PTMs and these mechanisms likely act in concert with one another as we previously described (Anderson et al., 2018b).

**FIGURE 1:**
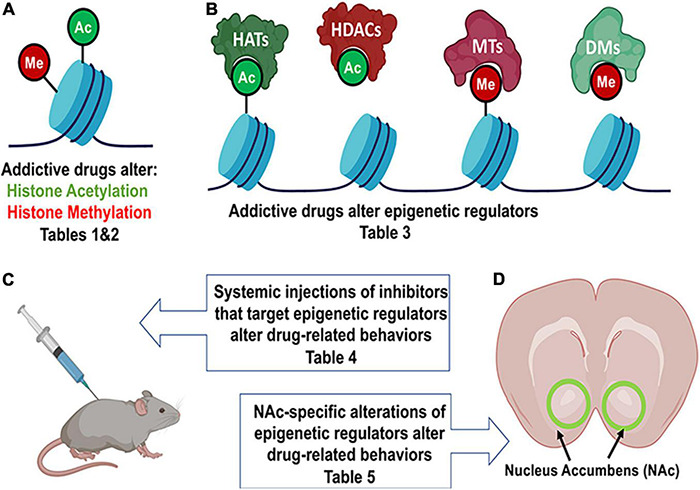
Drug exposure induces changes in epigenetic regulation in the nucleus accumbens and alters behavior. **(A)** Histone modifications are a form of epigenetic regulation that can alter DNA transcription. Two well studied modifications are acetylation (Ac) and methylation (Me). Increases in acetylation typically increase transcription and are thought to be “go” signals (green). In contrast, increases in methylation typically decrease transcription and are considered “stop” signals (red). Drug exposure has been shown to cause changes in histone acetylation (Table 1) and histone methylation (Table 2). **(B)** Histone acetylation levels of histones can be increased by histone acetyltransferases (HATs) and decreased by histone deacetylases (HDACs). Histone methylation levels can be increased by methyltransferases (MTs) and reduced by demethylases (DMs). Epigenetic proteins that modify histone acetylation and histone methylation are altered by drug exposure (Table 3). **(C)** Altering the activity of epigenetic proteins via systemic injection of inhibitor or activator compounds can alter drug-related behaviors in rodent models (Table 4). **(D)** Finally, accumbens-specific manipulations of epigenetic proteins can also alter drug-related behaviors (Table 5). This figure was created in part with biorender.com.

Importantly, in this review, we will focus on histone acetylation and histone methylation (Figure 1), but DNA methylation and chromatin structure are also altered by drug exposure in rodent models (Deng et al., 2010, 2014; Massart et al., 2015; Werner et al., 2021).

## Epigenetic Regulation in the Nucleus Accumbens

The NAc is part of the endogenous reward system and is critically involved in behavioral effects of addictive drugs. Drug exposure activates this system acutely but overtime are hypothesized to “hijack” this circuitry to increase drug-seeking (Nesse and Berridge, 1997). Many studies have examined epigenetic regulation in the NAc and found that many changes in histone acetylation and histone methylation occur after exposure to drugs (Anderson et al., 2018b; Werner et al., 2021). Though many areas in the brain show drug-induced epigenetic regulation like the dorsal striatum (Li et al., 2018), central nucleus of the amygdala, and orbitofrontal cortex (Cates et al., 2018), this review will only focus on the nucleus accumbens (NAc) (Figure 1). Here we summarize the literature in several tables that can be sorted by drug, type of administration (acute, chronic, or self-administered) or by the various drug-induced change in histone acetylation (Table 1) and histone methylation (Table 2) marks reported.

**TABLE 1:** Effects of drug exposure on histone acetylation.

Histone Target	Drug	Effect	Approach	Tissue collection timing	Type of administration	Drug administration	References	PMID
H2K12ac	Cocaine	↑ Acetylation	IHC with anti H2K12ac	1 hrs	Acute and Chronic	Cocaine (10 mg/kg, i.p.)	Malvaez et al., 2011	22114264
H3ac	Cocaine	↑ Acetylation at FosB promoter	ChIP-qPCR	1 hrs	Chronic	Cocaine (20 mg/kg, i.p.) for 7 days	Kumar et al., 2005	16242410
H3ac	Cocaine	↑ Acetylation at BDNF Promoter 2 and Cdk5 promoter	ChIP-qPCR	24 hrs	Chronic	Cocaine (20 mg/kg, i.p.) for 7 days	Kumar et al., 2005	16242410
H3ac	Cocaine	↑ Acetylation at 1004 promoters	ChIP on chip	24 hrs	Chronic	Cocaine (20 mg/kg, i.p.) for 7 days	Renthal et al., 2009	19447090
H3ac	Cocaine	↓ Acetylation at 83 promoters	ChIP on chip	24 hrs	Chronic	Cocaine (20 mg/kg, i.p.) for 7 days	Renthal et al., 2009	19447090
H3ac	Cocaine	↑ Acetylation at SIRT1, SIRT2 promoters	ChIP-qPCR	24 hrs	Chronic	Cocaine (20 mg/kg, i.p.) for 7 days	Renthal et al., 2009	19447090
H3ac	Cocaine	↑ Acetylation at BDNF Promoter 2	ChIP-qPCR	7 days	Chronic	Cocaine (20 mg/kg, i.p.) for 7 days	Kumar et al., 2005	16242410
H3ac	Cocaine	↑ Acetylation BDNF Promoter 2 and Cdk5 promoter	ChIP-qPCR	24 hrs	SA	Cocaine SA	Kumar et al., 2005	16242410
H3ac	Cocaine	↑ Acetylation at FosB promoter	ChIP-qPCR	24 hrs	SA	Cocaine SA	Kumar et al., 2005	16242410
H3ac	Cocaine	↑ Acetylation	WB anti H3Kac	3-24 h	SA	Cocaine SA	Wang et al., 2010	20010550
H3ac	Cocaine	↑ Acetylation	WB anti H3Kac	3-24 h	SA	Cocaine SA	Wang et al., 2010	20010550
H3ac	Cocaine	↑ Acetylation at BDNF-P2, BDNF-P3, FosB, Cdlk5, CaMKIIα, GluR2, NR2A, NR2B, and Psd95.	ChIP-qPCR		SA	Cocaine SA	Wang et al., 2010	20010550
H3K14ac	Cocaine	↑ Acetylation	IHC with anti H3K14ac	1 h	Acute and Chronic	Cocaine (10 mg/kg, i.p.)	Malvaez et al., 2011	22114264
H3K14ac	Cocaine	↑ Acetylation	nano LC-MS/MS			male progeny of cocaine sires	Wimmer et al., 2019	30565761
H3K18ac	Cocaine	↓ Acetylation	nano LC-MS/MS			male progeny of cocaine sires	Wimmer et al., 2019	30565761
H3K27ac	Cocaine	↑ Acetylation at Carpt promoter	ChIP-qPCR	24 h	Chronic	Cocaine (20 mg/kg, i.p.) for 10 days	Carpenter et al., 2020	31980629
H3K27ac	Cocaine	↑ Acetylation at Nr4a1 and Carpt promoter	ChIP-qPCR	28 days	Chronic	Cocaine (20 mg/kg, i.p.) for 10 days	Carpenter et al., 2020	31980629
H3K9/K14ac	Cocaine	↑ Acetylation	WB anti H3K9K14ac	0.5 h	Acute	Cocaine (20 mg/kg) with 3 CPP conditioning sessions	Li Y. et al., 2015	26377474
H3K9/K14ac	Cocaine	↑ Acetylation at CaMKIIα and Cdk5 promoter	ChIP-qPCR	24 h	Chronic	Cocaine (20 mg/kg, i.p.) for 7 days	Li Y. et al., 2015	26377474
H3K9ac	Cocaine	↑ Acetylation	WB with H3K9ac	24 h	Chronic	Cocaine (20 mg/kg, i.p.) for 7 days	Kennedy et al., 2013	23475113
H4ac	Cocaine	↑ Acetylation at cFos promoter	ChIP-qPCR	0.5 h and 1.5 h	Acute	Cocaine (20 mg/kg, i.p.)	Kumar et al., 2005	16242410
H4ac	Cocaine	↑ Acetylation at FosB promoter	ChIP-qPCR	1 h	Acute	Cocaine (20 mg/kg, i.p.)	Kumar et al., 2005	16242410
H4ac	Cocaine	↑ Acetylation at 692 promoters	ChIP on chip	24 h	Chronic	Cocaine (20 mg/kg, i.p.) for 7 days	Renthal et al., 2009	19447090
H4ac	Cocaine	↓ Acetylation at 123 promoters	ChIP on chip	24 h	Chronic	Cocaine (20 mg/kg, i.p.) for 7 days	Renthal et al., 2009	19447090
H4ac	Cocaine	↑ Acetylation	WB anti H4Kac	3-24 h	SA	Cocaine SA	Wang et al., 2010	20010550
H4ac	Cocaine	↑ Acetylation	IHC anti H4Kac	3-24 h	SA	Cocaine SA	Wang et al., 2010	20010550
H4ac	Cocaine	↑ Acetylation	IHC anti H4Kac	3-24 h	SA	Cocaine SA	Wang et al., 2010	20010550
H4ac	Cocaine	↑ Acetylation at Egr1 promoter	ChIP-qPCR		SA	Cocaine SA	Wang et al., 2010	20010550
H4K12ac	Cocaine	↓ Acetylation	IHC with anti H4K12ac	1 h	Acute and Chronic	Cocaine (10 mg/kg, i.p.)	Malvaez et al., 2011	22114264
H4K16ac	Cocaine	↑ Acetylation at numerous promoters	ChIP-seq	24 h	Chronic	Cocaine (20 mg/kg, i.p.) for 7 days	Ferguson et al., 2015	25698746
H4K5-16ac	Cocaine	↑ Acetylation at FosB promoter	ChIP-qPCR	0.3 h	Acute	Cocaine (30 mg/kg, i.p.)	Levine et al., 2011	22049069
H4K8ac	Cocaine	↑ Acetylation at Fos and Nr4a2 promoters	ChIP-qPCR	0.5 h	Acute	Cocaine (5 mg/kg) with CPP conditioning sessions	Rogge et al., 2013	23575859
H4K8ac	Cocaine	↑ Acetylation at Nr4a1 promoter	ChIP-qPCR	1 h	Chronic	Cocaine (20 mg/kg, i.p.) for 7 days	Campbell et al., 2021	33602824
phospho-H3ac	Cocaine	↑ Acetylation at cFos promoter	ChIP-qPCR	0.5 h and 1.5 h	Acute	Cocaine (20 mg/kg, i.p.)	Kumar et al., 2005	16242410
H3ac	Cocaine + stress	↑ Acetylation at BDNF promoter 1	ChIP-qPCR	0.5 h	Chronic	Cocaine binge (15 mg/kg, every hour for 3h) for 2 weeks and Swim stress	Cleck et al., 2008	18677617
H3ac	Methamphe tamine	↑ Acetylation	WB anti H3ac	0 h	Acute	METH (1 mg/kg, s.c.) with three CPP conditioning sessions	Shibasaki et al., 2011	21781114
H3ac	Methamphe tamine	↑ Acetylation at Nrxn, Syp, Dlg4, Gria1, Grin2a, Grin2b, Camk2a, Creb, Cdk5 promoters	ChIP-qPCR	0 hrs	Acute	METH (1 mg/kg, s.c.) with three CPP conditioning sessions	Shibasaki et al., 2011	21781114
H3K18ac	Methamphe tamine	↓ Acetylation	WB anti H3K18ac	8-24 h	Acute	METH (20 mg/kg, i.p.)	Martin et al., 2012	22470541
H3K9ac	Methamphe tamine	↓ Acetylation	WB anti H3K9ac	1-24 h	Acute	METH (20 mg/kg, i.p.)	Martin et al., 2012	22470541
H4ac	Methamphe tamine	↑ Acetylation at Cdk5 promoter	ChIP-qPCR	0 h	Acute	METH (1 mg/kg, s.c.) with three CPP conditioning sessions	Shibasaki et al., 2011	21781114
H4K5ac	Methamphe tamine	↑ Acetylation	WB anti H3K5ac	1-24 h	Acute	METH (20 mg/kg, i.p.)	Martin et al., 2012	22470541
H4K8ac	Methamphe tamine	↑ Acetylation	WB anti H3K8ac	16-24 h	Acute	METH (20 mg/kg, i.p.)	Martin et al., 2012	22470541
H3K14ac	Ethanol	↑ Acetylation	WB	0 h	Chronic	Chronic intermittent alcohol vapor exposure	Finegersh et al., 2015	26300722
H3K27ac	Ethanol	↑ Acetylation	WB anti H3K27ac	18 h	Drinking	Ethanol (daily 10% Ethanol, 2 hrs per day) for 10 days, drinking bottle	Griffin et al., 2017	29109977
H3K27ac	Ethanol	↑ Acetylation at FosB promoter	ChIP-qPCR	18 h	Drinking	Ethanol (daily 10% Ethanol, 2 hrs per day) for 10 days, drinking bottle	Griffin et al., 2017	29109977
H3K9ac	Ethanol	↑ Acetylation	WB anti H3Kac	24 h	Acute	Ethanol (2.5 g/kg, i.p.) and Ethanol (2.0 g/kg. i.p.) test	Sprow et al., 2014	25130590
H3K9ac	Ethanol	↑ Acetylation	IHC anti H3Kac	48-96 h	Acute	Ethanol (2.5 g/kg, i.p.) and Ethanol (1.5 g/kg. i.p.) test	Sprow et al., 2014	25130590
H3K9ac	Ethanol	↑ Acetylation	WB with anti H4K9ac	0 h	Chronic	Chronic intermittent alcohol vapor exposure	Finegersh et al., 2015	26300722
H3K9ac	Ethanol	↑ Acetylation	WB anti H3K9ac	24 h	Chronic	Ethanol (3 mg/kg, i.p.) for 8th injection	Pascual et al., 2009	19077056
H4ac	Ethanol	↑ Acetylation	IHC with anti H4ac	0.5 h	Chronic	Ethanol (2 g/kg, i.p.) for 10 days	Botia et al., 2012	23110077
H4ac	Ethanol	↓ Acetylation	WB anti H4ac	0 h	Drinking	Ethanol (20%) access in drinking water	Warnault et al., 2013	23423140
H4ac	Ethanol	↓ Acetylation	WB anti H4ac	0 h	Drinking	Ethanol (20%) SA	Warnault et al., 2013	23423140
H4K12ac	Ethanol	↑ Acetylation	WB anti H4K12ac	24 h	Chronic	Ethanol (3 mg/kg, i.p.) for 8th injection	Pascual et al., 2009	19077056
H3K18ac	Heroin	↑ Acetylation	IHC with anti H3K18ac	2 h	SA	Heroin SA + extinction 14 days + 2hrs after prime-reinstatement	Chen et al., 2016	27742468
H4K5ac	Heroin	↑ Acetylation	IHC with anti H4K5ac	2 h	SA	Heroin SA + extinction 14 days + 2hrs after prime-reinstatement	Chen et al., 2016	27742468
H4K8ac	Heroin	↑ Acetylation	IHC with anti H4K8ac	2 h	SA	Heroin SA + extinction 14 days + 2hrs after prime-reinstatement	Chen et al., 2016	27742468
H3K14ac	Morphine	↓ Acetylation	IHC with H3K14ac	1 h	Chronic	Chronic morphine (10-60 mg/kg) + naloxone (4 mg/kg)	Ciccarelli et al., 2013	23347952
H3K9ac	MDMA	↓ Acetylation at pN/OFQ promoter	pN/OFQ promoter	2 h	Acute	MDMA (8 mg/kg, i.p.)	Caputi et al., 2016	27989838
H3K9ac	MDMA	↑ Acetylation at proDynorphin promoter	proDynorphin promoter	2 h	Acute	MDMA (8 mg/kg, i.p.)	Caputi et al., 2016	27989838
H3K9ac	MDMA	↓ Acetylation at pN/OFQ promoter	pN/OFQ promoter	2 h	Chronic	MDMA (8 mg/kg, i.p.) twice per day for 7 days	Caputi et al., 2016	27989838
H3K14ac	THC	↑ Acetylation	WB	24 h	Chronic	THC (2.5, 5.0 and 10.0 mg/kg, i.p.) twice per day, for 11 days	Prini et al., 2017	28976920
H3K14ac	THC	↓ Acetylation	WB	48 h	Chronic	THC (2.5, 5.0 and 10.0 mg/kg, i.p.) twice per day, for 11 days	Prini et al., 2017	28976920
H3ac	Toluene	↑ Acetylation	IHC with antiH3ac	1 h	Chronic	Toluene (6000 ppm, 30 min exposure), twice a day for 10 sesession.	Sanchez-Serrano et al., 2011	21146589
H3K9ac	Nicotine	↑ Acetylation	WB anti H3K9ac	0.3 h	Chronic	Nicotine (10 mg/ml) in drinking water for 7 days	Levine et al., 2011	22049069
H3K9ac	Nicotine	↑ Acetylation at FosB promoter	ChIP-qPCR	0.3 h	Chronic	Nicotine (10 mg/ml) in drinking water for 7 days	Levine et al., 2011	22049069
H4K5-16ac	Nicotine	↑ Acetylation	WB anti H4K5toK16ac	0.3 h	Chronic	Nicotine (10 mg/ml) in drinking water for 7 days	Levine et al., 2011	22049069
H4K5-16ac	Nicotine	↑ Acetylation at FosB promoter	ChIP-qPCR	0.3 h	Chronic	Nicotine (10 mg/ml) in drinking water for 7 days	Levine et al., 2011	22049069

**TABLE 2:** Effects of drug exposure on histone methylation.

Histone Target	Drug	Effect	Approach	Tissue collection timing	Type of administration	Drug administration	References	PMID
H3K20me2	Cocaine	↓ Methylation	nanoLC-MSMS	Offspring	SA		Wimmer et al., 2019	30565761
H3K27me2	Cocaine	↓ Methylation	nanoLC-MSMS	Offspring	SA		Wimmer et al., 2019	30565761
H3K27me3	Cocaine	↓ Methylation at pDYN promoter	ChIP-qPCR	0 hrs	Chronic	Cocaine (50 mg/kg per day via pumps) for 7 days	Caputi et al., 2014	24184686
H3K27me3	Cocaine	↓ Methylation at NOP promoter	ChIP-qPCR	0 hrs	Chronic	Cocaine (50 mg/kg per day via pumps) for 7 days	Caputi et al., 2014	24184686
H3K27me3	Cocaine	↑ Methylation at Cartpt promoter	ChIP-qPCR	24 hrs	Chronic	Cocaine (20 mg/kg, i.p.) for 10 days	Carpenter et al., 2020	31980629
H3K27me3	Cocaine	Numerous changes	ChIP-seq	24 hrs	Chronic	Cocaine (20 mg/kg, i.p.) for 7 days	Feng et al., 2014	24758366
H3K27me3	Cocaine	↓ Methylation at Cartpt promoter	ChIP-qPCR	28 days	Chronic	Cocaine (20 mg/kg, i.p.) for 10 days	Carpenter et al., 2020	31980629
H3K36me3	Cocaine	Numerous changes	ChIP-seq	24 hrs	Chronic	Cocaine (20 mg/kg, i.p.) for 7 days	Feng et al., 2014	24758366
H3K36me3	Cocaine	↓ Methylation	WB	24 hrs	Chronic	Cocaine (20 mg/kg, i.p.) for 7 days	Li Y. et al., 2015	26377474
H3K4me1	Cocaine	Numerous changes	ChIP-seq	24 hrs	Chronic	Cocaine (20 mg/kg, i.p.) for 7 days	Feng et al., 2014	24758366
H3K4me2	Cocaine	↓ Methylation	nanoLC-MSMS	Offspring	SA		Wimmer et al., 2019	30565761
H3K4me3	Cocaine	↑ Methylation at NOP promoter	ChIP-qPCR	0 hrs	Chronic	Cocaine (50 mg/kg per day via pumps) for 7 days	Caputi et al., 2014	24184686
H3K4me3	Cocaine	↓ Methylation at pN/OFQ promoter	ChIP-qPCR	0 hrs	Chronic	Cocaine (50 mg/kg per day via pumps) for 7 days	Caputi et al., 2014	24184686
H3K4me3	Cocaine	↑ Methylation at Cartpt promoter	ChIP-qPCR	24 hrs	Chronic	Cocaine (20 mg/kg, i.p.) for 10 days	Carpenter et al., 2020	31980629
H3K4me3	Cocaine	↑ Methylation at PGC-1a promoter	ChIP-qPCR	24 h	Chronic	Cocaine (20 mg/kg, i.p.) for 7 days	Chandra et al., 2017	27939396
H3K4me3	Cocaine	Numerous changes	ChIP-seq	24 h	Chronic	Cocaine (20 mg/kg, i.p.) for 7 days	Feng et al., 2014	24758366
H3K4me3	Cocaine	↑ Methylation at Nr4a1 and Cartpt	ChIP-qPCR	28 days	Chronic	Cocaine (20 mg/kg, i.p.) for 10 days	Carpenter et al., 2020	31980629
H3K9/K27me2	Cocaine	↑ Mehylation at 898 promoters	ChIP on chip	24 h	Chronic	Cocaine (20 mg/kg, i.p.) for 7 days	Renthal et al., 2009	19447090
H3K9/K27me2	Cocaine	↓ Methylation at 209 promoters	ChIP on chip	24 h	Chronic	Cocaine (20 mg/kg, i.p.) for 7 days	Renthal et al., 2009	19447090
H3K9me2	Cocaine	↓ Methylation	IHC	1 h	Acute	Cocaine (10 mg/kg, i.p.)	Malvaez et al., 2011	22114264
H3K9me2	Cocaine	↓ Methylation at FosB promoter	ChIP-qPCR	1 h	Chronic	Cocaine (15 mg/kg, i.p) for 10 days, withdrawal 28 days + Challenge Cocaine (15 mg/kg)	Damez-Werno et al., 2012	22836260
H3K9me2	Cocaine	↓ Methylation	IHC	1 h	Chronic	Cocaine (10 mg/kg, i.p.) for 5 days	Malvaez et al., 2011	22114264
H3K9me2	Cocaine	↓ Methylation	WB	24 h	Chronic	Cocaine (20 mg/kg, i.p.) for 7 days	Covington et al., 2011	21867882
H3K9me2	Cocaine	Numerous changes	ChIP-seq	24 h	Chronic	Cocaine (20 mg/kg, i.p.) for 7 days	Feng et al., 2014	24758366
H3K9me2	Cocaine	↓ Methylation at the Gabrb3	ChIP-qPCR	24 h	Chronic	Cocaine (20 mg/kg, i.p.) for 7 days	Kennedy et al., 2013	23475113
H3K9me2	Cocaine	↓ Methylation	WB	24 h	Chronic	Cocaine (20 mg/kg, i.p.) for 7 days	Li Y. et al., 2015	26377474
H3K9me2	Cocaine	↓ Methylation	WB	24 h	Chronic	Cocaine (20 mg/kg, i.p.) for 7 days	Maze et al., 2010	20056891
H3K9me2	Cocaine	↓ Methylation at Cdk5, p65/NFkB, Arc, FosB, LIMK, BDNF, APRT promoters	ChIP-qPCR	24 h	Chronic	Cocaine (20 mg/kg, i.p.) for 7 days	Maze et al., 2010	20056891
H3K9me2	Cocaine	↓ Methylation at FosB promoter	ChIP-qPCR	28 Days	Chronic	Cocaine (15 mg/kg, i.p.) for 10 days	Damez-Werno et al., 2012	22836260
H3K9me2	Cocaine	↓ Methylation at D2 promoter	ChIP-PCR	30 days	SA	Cocaine SA, acquisiion for 60 days, drug reinstatement at 72 days, and brain collection on 170 day	Flagel et al., 2016	27114539
H3K9me3	Cocaine	↑ Methylation	WB	0.5 h	Acute	Cocaine (20 mg/kg, i.p.)	Maze et al., 2011	21300862
H3K9me3	Cocaine	↑ Methylation	WB	1 h	Acute	Cocaine (20 mg/kg, i.p.)	Maze et al., 2011	21300862
H3K9me3	Cocaine	↑ Methylation	WB	1 h	Chronic	Cocaine (20 mg/kg, i.p.) for 7 days	Maze et al., 2011	21300862
H3K9me3	Cocaine	↑ Methylation at Auts2 and Caln1 promoters	ChIP-qPCR	24 h	Chronic	Cocaine (20 mg/kg, i.p.) for 7 days	Engmann et al., 2017	28577753
H3K9me3	Cocaine	Numerous changes	ChIP-seq	24 h	Chronic	Cocaine (20 mg/kg, i.p.) for 7 days	Feng et al., 2014	24758366
H3K9me3	Cocaine	↓ Methylation at numerous sites, 32,956 and 30,412 peaks in saline- and cocaine-treated animals,	ChIP-seq	24 h	Chronic	Cocaine (20 mg/kg, i.p.) for 7 days	Maze et al., 2011	21300862
H3K9me3	Cocaine	↓ Methylation	WB	7 days	Chronic	Cocaine (20 mg/kg, i.p.) for 7 days	Maze et al., 2011	21300862
H3R2me2a	Cocaine	↑ Methylation at 208 sites	ChIP-seq	24 h	Chronic	Cocaine (20 mg/kg, i.p.) for 7 days	Damez-Werno et al., 2016	27506785
H3R2me2a	Cocaine	↓ Methylation	WB	24 h	Chronic	Cocaine (20 mg/kg, i.p.) for 7 days	Damez-Werno et al., 2016	27506785
H3R2me2a	Cocaine	↓ Methylation at 129 site	ChIP-seq	24 h	Chronic	Cocaine (20 mg/kg, i.p.) for 7 days	Damez-Werno et al., 2016	27506785
H3R2me2a	Cocaine	↓ Methylation	WB	7 days	SA	Cocaine SA + 7 days withdrawal	Damez-Werno et al., 2016	27506785
H4R3me2a	Cocaine	↑ Methylation	WB	0.5 h	Acute	Cocaine (20 mg/kg) with 3 CPP conditioning	Li Y. et al., 2015	26377474
H4R3me2a	Cocaine	↑ Methylation	WB	24 h	Chronic	Cocaine (20 mg/kg, i.p.) for 7 days	Li Y. et al., 2015	26377474
H4R3me2a	Cocaine	↑ Methylation, CaMKIIα and Cdk5 promoter	ChIP-qPCR	24 h	Chronic	Cocaine (20 mg/kg, i.p.) for 7 days	Li Y. et al., 2015	26377474
H4R3me2a	Cocaine	↑ Methylation	WB	7 days	Chronic	Cocaine (20 mg/kg, i.p.) for 7 days	Li Y. et al., 2015	26377474
H3K27me3	Methamphe tamine	↓ Methylation	WB	1.5 h	Acute	Meth with CPP 30 min conditining	Aguilar-Valles et al., 2014	24183790
H3K4me2	Methamphe tamine	↑ Methylation	WB	1.5 h	Acute	Meth with CPP 30 min conditining	Aguilar-Valles et al., 2014	24183790
H3K4me3	Methamphe tamine	↑ Methylation at Oxtr promoter	ChIP-qPCR	1.5 h	Acute	Meth with CPP 30 min conditining	Aguilar-Valles et al., 2014	24183790
H3K4me3	Methamphe tamine	↑ Methylation at CCR2 promoter	ChIP-qPCR	24 h	Chronic	Meth (2 mg/kg, s.c.) 5 intermittent treatment with once every 96 hrs	Ikegami et al., 2010	20624155
H3K9me2	Amphe tamine	↑ Methylation at c-fos promoter	ChIP-qPCR	5 days	Chronic	Amphetamine (4 mg/kg, i.p.) for 7 days	Renthal et al., 2008	18632938
H3K27me3	Ethanol	↑ Methylation, 3 peaks	ChIP-seq	3 weeks	Chronic	Chronic intermittent alcohol vapor exposure	Johnstone et al., 2021	31373129
H3K27me3	Ethanol	↓ Methylation	WB	3 weeks	Chronic	Chronic intermittent alcohol vapor exposure	Johnstone et al., 2021	31373129
H3K27me3	Ethanol	↓ Methylation, 90 peaks	ChIP-seq	3 weeks	Chronic	Chronic intermittent alcohol vapor exposure	Johnstone et al., 2021	31373129
H3K9me2	Ethanol	↓ Methylation	WB	3d	Chronic	Chronic intermittent alcohol vapor exposure	Anderson et al., 2021	34013595
H3K4me3	Morphine	↑ Methylation at Sirt1	ChIP-seq	24 h	Chronic	Morphine (20 mg/kg, i.p.) for 7 days	Ferguson et al., 2013	24107942
H3K9me2	Morphine	↑ Methylation 5666 promoters	ChIP-seq	24 h	Chronic	Morphine (20 mg/kg, i.p.) for 7 days	Sun et al., 2012	23197736
H3K9me2	Morphine	↑ Methylation at Gria1 promoter	ChIP-qPCR	24 h	Chronic	Morphine (20 mg/kg, i.p.) for 7 days	Sun et al., 2012	23197736
H3K9me2	Morphine	↓ Methylation	WB	24 h	Chronic	Morphine (20 mg/kg, i.p.) for 5 and 7 days	Sun et al., 2012	23197736
H3K9me2	Morphine	↓ Methylation 8106 promoters	ChIP-seq	24 h	Chronic	Morphine (20 mg/kg, i.p.) for 7 days	Sun et al., 2012	23197736
H3K9me2	Morphine	↓ Methylation at Grin2a, Grm5, Grm8 promoters	ChIP-qPCR	24 h	Chronic	Morphine (20 mg/kg, i.p.) for 7 days	Sun et al., 2012	23197736
H3K27me3	MDMA	↑ Methylation at pDYN promoter	ChIP-qPCR	2 h	Chronic	MDMA (8 mg/kg, i.p.) twice per day for 7 days	Caputi et al., 2016	27989838
H3K4me3	MDMA	↑ Methylation at NOP promoter	ChIP-qPCR	2 h	Acute	MDMA (8 mg/kg, i.p.)	Caputi et al., 2016	27989838
H3K4me3	MDMA	↑ Methylation at pDYN promoter	ChIP-qPCR	2 h	Acute	MDMA (8 mg/kg, i.p.)	Caputi et al., 2016	27989838
H3K4me3	MDMA	↑ Methylation at pN/OFQ promoter	ChIP-qPCR	2 h	Acute	MDMA (8 mg/kg, i.p.)	Caputi et al., 2016	27989838
H3K9me2	MDMA	↓ Methylation at pDYN promoter	ChIP-qPCR	2 h	Acute	MDMA (8 mg/kg, i.p.)	Caputi et al., 2016	27989838
H3K4me3	THC	↑ Methylation at Penk gene	ChIP-qPCR	24 h	Chronic	THC (1.5 mg/kg) for every three days (8 injections) in adolescent	Tomasiewicz et al., 2012	22683090
H3K9me2	THC	↑ Methylation	WB	24 h	Chronic	THC (2.5, 5.0 and 10.0 mg/kg, i.p.) twice per day, for 11 days	Prini et al., 2017	28976920
H3K9me2	THC	↓ Methylation at Penk gene	ChIP-qPCR	24 h	Chronic	THC (1.5 mg/kg) for every three days (8 injections) in adolescent	Tomasiewicz et al., 2012	22683090
H3K9me2	THC	↓ Methylation at Penk gene	ChIP-qPCR	30 days	Chronic	THC (1.5 mg/kg) for every three days (8 injections) in adolescent	Tomasiewicz et al., 2012	22683090
H3K9me2	THC	↓ Methylation	WB	48 h	Chronic	THC (2.5, 5.0 and 10.0 mg/kg, i.p.) twice per day, for 11 days	Prini et al., 2017	28976920
H3K9me3	THC	↑ Methylation	WB	2 h	Chronic	THC (2.5, 5.0 and 10.0 mg/kg, i.p.) twice per day, for 11 days	Prini et al., 2017	28976920
H3K9me3	THC	↑ Methylation	WB	24 h	Chronic	THC (2.5, 5.0 and 10.0 mg/kg, i.p.) twice per day, for 11 days	Prini et al., 2017	28976920
H3K9me3	THC	↓ Methylation at Penk gene	ChIP-qPCR	30 days	Chronic	THC (1.5 mg/kg) for every three days (8 injections) in adolescent	Tomasiewicz et al., 2012	22683090
H3K9me3	THC	↓ Methylation	WB	48 h	Chronic	THC (2.5, 5.0 and 10.0 mg/kg, i.p.) twice per day, for 11 days	Prini et al., 2017	28976920

### Effects of Drug Exposure on Histone Acetylation

Many addictive drugs cause changes in histone acetylation, including cocaine, methamphetamine, ethanol, opioids, MDMA, THC, toluene, and nicotine.

#### Cocaine

Cocaine exposure alters many histone acetylation marks in the NAc (Table 1). Cocaine exposure typically increases global acetylation of the histones H3 and H4 in the NAc, likely by increasing individual sites like H2K12, H3K9, H3K14, H3K27, H4K5, H4K8, and H4K16 (Kumar et al., 2005; Cleck et al., 2008; Renthal et al., 2009; Wang et al., 2010; Levine et al., 2011; Malvaez et al., 2011; Kennedy et al., 2013; Rogge et al., 2013; Ferguson et al., 2015; Li Y. et al., 2015; Wimmer et al., 2019; Carpenter et al., 2020; Campbell et al., 2021). Some of these changes in acetylation reflect global changes from whole NAc tissue, but others reflect specific changes at certain promoters (see Table 1 for details). Cocaine-induced decreases in acetylation have also been reported for H3ac, H3K18, H4ac, and H4K12 (Renthal et al., 2009; Malvaez et al., 2011; Wimmer et al., 2019). Cocaine can alter histone acetylation very quickly, but can also produce long-lasting changes as the findings reflect a range of timepoints following the last exposure to cocaine from 20 min to 28 days (Levine et al., 2011; Carpenter et al., 2020). Importantly, while most of these studies used experimenter (non-contingent) exposure, self-administered (contingent) cocaine similarly increases acetylation of histone H3 and H4 at certain promoters 3-24 h after the last self-administration (Kumar et al., 2005; Wang et al., 2010). These data show that exposure to cocaine rapidly changes histone acetylation in many gene promoters, and at least some of these changes can last up to 28 days later.

#### Methamphetamine

Non-contingent methamphetamine exposure increases pan-H3 acetylation, H4ac at a specific promoter, H4K5, and H4K8 (Shibasaki et al., 2011; Martin et al., 2012). Non-contingent methamphetamine also decreases H3K9 and H3K18 up to 24 hrs later (Martin et al., 2012).

#### Ethanol

Non-contingent ethanol exposure increases pan-H4 acetylation, and the specific marks H3K14, H3K9, and H4K12 (Pascual et al., 2009; Botia et al., 2012; Sprow et al., 2014; Finegersh et al., 2015). In contrast, self-administered ethanol reduces pan-H4 acetylation (Warnault et al., 2013) and increases H3K27 acetylation (Griffin et al., 2017). These findings suggest that at least some differences (pan-H4 acetylation) are found between contingent and non-contingent rodent models of SUD.

#### Opioids

Heroin primed reinstatement of drug-seeking behavior following heroin self-administration increases acetylation of H3K18, H4K5, and H4K8 (Chen et al., 2016). Naloxone-precipitated withdrawal administration after chronic non-contingent morphine exposure reduces H3K14 acetylation in the NAc shell (Ciccarelli et al., 2013).

#### MDMA

Non-contingent MDMA changes H3K9 acetylation at specific promoters (Caputi et al., 2016).

#### THC

Non-contingent THC increases H3K14ac at 24hrs after the last exposure but then decreases by 48 hrs after the last exposure (Prini et al., 2017).

#### Toluene

Chronic non-contingent exposure to toluene increases pan-H3 acetylation in the NAc (Sanchez-Serrano et al., 2011).

#### Nicotine

Chronic nicotine exposure for 7 days through drinking water increases the acetylation level of Histone H3K9 and H4K5-K16 (Levine et al., 2011).

Combined, these studies suggest that most psychoactive, addictive drugs alter histone acetylation in the NAc and highlight that some of these changes may be short-lived and very dynamic (Prini et al., 2017).

### Effects of Drugs Exposure on Histone Methylation

The nucleus accumbens also undergoes changes in methylated histone marks following exposure to addictive drugs like cocaine, methamphetamine, ethanol, opioids, MDMA, and THC (Table 2).

#### Cocaine

Non-contingent cocaine exposure alters many histone methylation sites including H3K27me3, H3K36me3, H3K4me1, H3K4me2, H3K4me3, H3K9/K27me2, H3K9me2, H3K9me3, H3R2me2a, H4K9me3, and H4R3me2a (Adams and Bushell, 1989; Renthal et al., 2009; Maze et al., 2010, 2011; Covington et al., 2011; Malvaez et al., 2011; Damez-Werno et al., 2012, 2016; Kennedy et al., 2013; Caputi et al., 2014; Feng et al., 2014; Li Y. et al., 2015; Chandra et al., 2017; Engmann et al., 2017; Carpenter et al., 2020). These changes include both increases and decreases of methylation at these histone sites (see Table 2 for details on each study). In addition to non-contingent rodent models of SUD, contingent cocaine decreases methylation of H3K9me2 at specific promoters and pan-H3R2me2a as well (Damez-Werno et al., 2016; Wimmer et al., 2019).

#### Methamphetamine and Amphetamine

Non-contingent methamphetamine exposure decreases H3K27me3 methylation, but increases H3K4me2 and H3K4me3 methylation. Some promoter specific changes remain for at least 24 h (Ikegami et al., 2010; Aguilar-Valles et al., 2014). Amphetamine increases H3K9 methylation on the fos promoter 5 days after the last exposure (Renthal et al., 2008).

#### Ethanol

Non-contingent alcohol exposure by the chronic intermittent ethanol vapor exposure model alters H3K27me3 and decreases H3K9me2 (Anderson et al., 2021; Johnstone et al., 2021).

#### Opioids

Non-contingent morphine exposure causes both increases and decreases in H3K9me2 and H3K4me3 that are promoter specific (Sun et al., 2012; Ferguson et al., 2013).

#### MDMA

Non-contingent MDMA increases H3K27me3 and H3K4me3 at specific promoters and decreases H3K9me2 at others (Caputi et al., 2016).

#### THC

Non-contingent THC alters H3K9me2 and H3K4me3 levels, some at specific promoters (Tomasiewicz et al., 2012; Prini et al., 2017). Of note, THC causes bidirectional changes in H3K9me2 over 1 vs 2 days after the last exposure (Prini et al., 2017) suggesting some of these histone changes may be very short-lived and highly dynamic. Also, some of these THC-induced changes last up to 30 days later at specific promoters (Tomasiewicz et al., 2012).

### General Conclusions on Drug-Altered Histone Post-translational Modifications

Some similar general conclusions can be drawn when examining both histone acetylation and histone methylation following exposure to drugs exposure (Tables 1, 2).

First, different drugs cause different changes in the histone marks. This suggests no clear common “histone code” for drug exposure in the NAc. For instance, cocaine, ethanol, and THC lead to increases in H3K14ac cocaine (Malvaez et al., 2011; Kennedy et al., 2013; Finegersh et al., 2015; Prini et al., 2017), but morphine leads to a decrease (Ciccarelli et al., 2013). Differences in study design and timepoints could affect these findings, for instance, H3K14ac is increased 24 h after THC, but reduced 48 h later (Prini et al., 2017).

Second, most of these histone PTM changes are present at very early timepoints following the last exposure to an addictive drug. In addition, these histone acetylation and histone methylation changes appear to occur very rapidly - even after an acute dose (Martin et al., 2012; Godino et al., 2015) - and are likely highly dynamic or short-lived. In other words, there are large signaling changes in epigenetic marks shortly after the last drug exposure, but most of these changes appear to return to baseline levels following longer timescales.

Third, a small subset of changes at certain gene promoters appear to persist for longer periods of time after the last drug exposure. For instance, increased H3 acetylation at the BDNF promoter (an important mediator of drug-related behaviors (Graham et al., 2007; Bahi et al., 2008; Lobo et al., 2010; Li et al., 2013; Anderson et al., 2017) is observed after 7d withdrawal (Kumar et al., 2005) and increased methylation of H4R3me2a is observed at both 1d and 7d (but not 14d) withdrawal from cocaine (Li Y. et al., 2015). Cocaine also leads to a stable decrease in H3K9me2 at the D2 promoter after a month of withdrawal in rats bred for high responding (Flagel et al., 2016). In addition, THC causes lasting changes at H3K9, as a decrease in methylation is observed at the proenkephalin gene promoter at both 1d and 30d withdrawal (Tomasiewicz et al., 2012). Also, chronic intermittent alcohol vapor exposure decreases H3K27me3 after 3 weeks of withdrawal (Johnstone et al., 2021). Finally, cocaine causes an increase in H3K27ac and H3K4me3 at the *cartpt* promoter that was found at both 1d and 28d of abstinence. This same study also found that H3K27me3 was increased after 1d of abstinence, but was reduced after 28d of abstinence (Carpenter et al., 2020). So, while all classes of drugs exposure led to short term changes in histone marks, at least some of these changes may remain for longer periods of time and could possibly cause long-lasting behavioral changes.

## Effects of Drugs on Epigenetic Regulators in the Nucleus Accumbens

Addictive drugs also cause changes to the proteins that regulate histone marks in the NAc, and this suggests that we can alter these drug-induced histone marks by targeting their epigenetic regulators. As shown in Table 3 there are many known candidates that are regulated by drug exposure in the NAc.

**TABLE 3:** Effects of drug exposure on epigenetic regulators in the NAc.

Epigenetic Target	Drug	Effect	Approach	Tissue collection timing	Type of administration	Drug administration	References	PMID
HDACs	Ethanol	↓ nuclear activity		22 hrs	Drinking	Ethanol (daily 10% Ethanol, 2 hrs per day) for 10 days	Griffin et al., 2017	29109977
HDACs	Nicotine	↓ activity		0 hrs	Drinking	Nicotine (10 mg/ml) in drinking water for 7-10 days	Levine et al., 2011	22049069
HDAC1	Amphe tamine	↑ enrichment on c-fos promoter	ChIP-qPCR	5 days	Chronic	Amphetamine (4 mg/kg, i.p.) for 7 days	Renthal et al., 2009	18632938
HDAC1	Cocaine	↑ binding to G9a and GLP promoters	ChIP-qPCR	4 h	Chronic	Cocaine (20 mg/kg, i.p.) for 7 days	Kennedy et al., 2013	23475113
HDAC1	Methamphe tamine	↓ protein	WB	1-16 h	Acute	METH (20 mg/kg, i.p.)	Martin et al., 2012	22470541
HDAC2	Cocaine	↓ protein association with PARP-1 complexes	WB	0.5 h	Chronic	Cocaine (20 mg/kg, i.p.) for 7 days	Scobie et al., 2014	24449909
HDAC2	Cocaine	↑ mRNA	qPCR	2 h	SA	Cocaine SA	Host et al., 2011	19939859
HDAC2	Cocaine	↑ protein	IHC	2 h	SA	Cocaine SA	Host et al., 2011	19939859
HDAC2	Ethanol	↑ mRNA	qPCR	18 h	SA	Chronic intermittent access two bottle choice 20% alcohol drinking 3 days per week for 4 weeks	Sharma et al., 2021	34837399
HDAC2	Methamphe tamine	↑ protein	WB	1-8 h	Acute	METH (20 mg/kg, i.p.)	Torres et al., 2015	26300473
HDAC2	Methamphe tamine	↑ binding to fosB, fra2, and Egr3 promoters	ChIP-qPCR	2 h	Acute	METH (20 mg/kg, i.p.)	Torres et al., 2015	26300473
HDAC2	Methamphe tamine	↑ protein	WB	4-24 h	Acute	METH (20 mg/kg, i.p.)	Martin et al., 2012	22470541
HDAC2	Nicotine	↑ protein		1 day	Acute	Nicotine (0.4 mg/kg, i.p.) with CPP 4 conditining	Faillace et al., 2015	25981209
HDAC3	Cocaine	↓ binding at promoters (Fos, Nr4a2)	ChIP-qPCR	1 h	Acute	Cocaine (5 mg/kg, i.p.) with CPP conditining	Rogge et al., 2013	23575859
HDAC3	Cocaine	↑ binding to Fos and Nr4a1 promoters	ChIP-qPCR	1 h	Chronic	Cocaine (20 mg/kg, i.p.) for 7 days	Campbell et al., 2021	33602824
HDAC3	Cocaine	↑ mRNA, in D1-MSN	qPCR	1 h	Chronic	Cocaine (20 mg/kg, i.p.) for 7 days	Campbell et al., 2021	33602824
HDAC3	Methamphe tamine	↓ mRNA	qPCR	1 h	Acute	METH (20 mg/kg, i.p.)	Torres et al., 2016	26721795
HDAC3	Methamphe tamine	↓ mRNA	qPCR	8 h	Acute	METH (20 mg/kg, i.p.)	Torres et al., 2016	26721795
HDAC4	Cocaine	↑ Nuclear Export	WB	4 h	Chronic	Cocaine (20 mg/kg, i.p.) for 7 days	Penrod et al., 2018	28635037
HDAC4	Cocaine	↑ phosphorylation	WB	4 h	Chronic	Cocaine (20 mg/kg, i.p.) for 7 days	Penrod et al., 2018	28635037
HDAC4	Ethanol	↓ protein	WB	18 h	Drinking	Ethanol (daily 10% Ethanol, 2 hrs per day) for 10 days	Griffin et al., 2017	29109977
HDAC4	Ethanol	↓ protein in the nuclear at 18 hrs	WB	18 h	Drinking	Ethanol (daily 10% Ethanol, 2 hrs per day) for 10 days	Griffin et al., 2017	29109977
HDAC4	Ethanol	↑ mRNA	RNA-seq	22 h	Drinking	Ethanol (daily 20% Ethanol, 2 hrs per day) for 6 weeks	Pozhidayeva et al., 2020	32085427
HDAC4	Methamphe tamine	↓ mRNA	qPCR	1 h	Acute	METH (20 mg/kg, i.p.)	Torres et al., 2016	26721795
HDAC4	Methamphe tamine	↓ mRNA	qPCR	2 h	Acute	METH (20 mg/kg, i.p.)	Torres et al., 2016	26721795
HDAC4	Methamphe tamine	↓ mRNA	qPCR	8 h	Acute	METH (20 mg/kg, i.p.)	Torres et al., 2016	26721795
HDAC5	Cocaine	↑ Nuclear Export	IHC	0.5 h	Chronic	Cocaine (20 mg/kg, i.p.) for 7 days	Renthal et al., 2007	17988634
HDAC5	Cocaine	↑ phosphorylation	WB	0.5 h	Chronic	Cocaine (20 mg/kg, i.p.) for 7 days	Renthal et al., 2007	17988634
HDAC5	Cocaine	↓ mRNA	qPCR	1 h	Acute	Cocaine (5 mg/kg, i.p.) with CPP conditining	Rogge et al., 2013	23575859
HDAC5	Cocaine	↓ phosphorylation	WB	1 h	Acute	Cocaine (20 mg/kg, i.p.)	Taniguchi et al., 2012	22243750
HDAC5	Cocaine	↓ phosphorylation	WB	1 h	Chronic	Cocaine (20 mg/kg, i.p.) for 7 days	Taniguchi et al., 2012	22243750
HDAC5	Cocaine	↓ nuclear localization	IHC	2 h	SA	Cocaine SA	Host et al., 2011	19939859
HDAC5	Cocaine	↑ nuclear Import	WB	4 h	Acute	Cocaine (20 mg/kg, i.p.)	Taniguchi et al., 2012	22243750
HDAC5	Cocaine	↓ phosphorylation	WB	4 h	Acute	Cocaine (20 mg/kg, i.p.)	Taniguchi et al., 2012	22243750
HDAC5	Cocaine	↑ nuclear Import	WB	4 h	Chronic	Cocaine (20 mg/kg, i.p.) for 7 days	Taniguchi et al., 2012	22243750
HDAC5	Cocaine	↓ phosphorylation	WB	4 h	Chronic	Cocaine (20 mg/kg, i.p.) for 7 days	Taniguchi et al., 2012	22243750
HDAC5	Ethanol	↓ mRNA	RNA-seq	22 h	Drinking	Ethanol (daily 20% Ethanol, 2 hrs per day) for 6 weeks	Pozhidayeva et al., 2020	32085427
HDAC5	Heroin	↓ mRNA, human	Microarray		Human	Heroin overdose	Egervari et al., 2017	27863698
HDAC6	Methamphe tamine	↑ mRNA	qPCR	1 h	Acute	METH (20 mg/kg, i.p.)	Torres et al., 2016	26721795
HDAC6	Methamphe tamine	↑ mRNA	qPCR	2 h	Acute	METH (20 mg/kg, i.p.)	Torres et al., 2016	26721795
HDAC6	Methamphe tamine	↑ mRNA	qPCR	8 h	Acute	METH (20 mg/kg, i.p.)	Torres et al., 2016	26721795
HDAC7	Methamphe tamine	↓ mRNA	qPCR	1 h	Acute	METH (20 mg/kg, i.p.)	Torres et al., 2016	26721795
HDAC7	Methamphe tamine	↓ mRNA	qPCR	2 h	Acute	METH (20 mg/kg, i.p.)	Torres et al., 2016	26721795
HDAC8	Methamphe tamine	↓ mRNA	qPCR	8 h	Acute	METH (20 mg/kg, i.p.)	Torres et al., 2016	26721795
HDAC9	Ethanol	↓ mRNA	NanoString analysis	3 weeks	Chronic	Chronic intermittent alcohol vapor exposure	Johnstone et al., 2021	31373129
HDAC11	Cocaine	↑ protein	IHC	2 h	SA	Cocaine SA	Host et al., 2011	19939859
HDAC11	Ethanol	↓ mRNA	qPCR	0.5 h	Acute	Ethanol (2 g/kg, i.p), challenge at 17 days	Botia et al., 2012	23110077
HDAC11	Ethanol	↓ mRNA, sensitized animals	qPCR	0.5 h	Chronic	Ethanol (2 g/kg, i.p), for 10 days + Ethanol challenge at 17 days	Botia et al., 2012	23110077
HDAC11	Ethanol	↑ mRNA in high drinkers		6 h	Drinking	Ethanol drinking sessions (4 drinking and 4 days of abstinence, repeated four times)	Wolstenholme et al., 2011	21698166
HDAC11	Methamphe tamine	↓ mRNA	qPCR	1 h	Acute	METH (20 mg/kg, i.p.)	Torres et al., 2016	26721795
HDAC11	Methamphe tamine	↓ mRNA	qPCR	2 h	Acute	METH (20 mg/kg, i.p.)	Torres et al., 2016	26721795
HDAC11	Methamphe tamine	↓ mRNA	qPCR	8 h	Acute	METH (20 mg/kg, i.p.)	Torres et al., 2016	26721795
SIRT1	Cocaine	↑ mRNA	qPCR	24 h	Chronic	Cocaine (20 mg/kg, i.p.) for 7 days	Ferguson et al., 2013	24107942
SIRT1	Cocaine	↑ Protein	WB	24 h	Chronic	Cocaine (20 mg/kg, i.p.) for 7 days	Ferguson et al., 2013	24107942
SIRT1	Cocaine	↓ SIRT1 binding to numerous promoters	ChIP-SIRT, 125 increase and 488 decrease in promoter after cocaine	24 h	Chronic	Cocaine (20 mg/kg, i.p.) for 7 days	Ferguson et al., 2015	25698746
SIRT1	Cocaine	↓ SIRT1 binding to numerous promoters	ChIP-SIRT, 8949 decrease and 2245 increase after cociane	24 h	Chronic	Cocaine (20 mg/kg, i.p.) for 7 days	Ferguson et al., 2015	25698746
SIRT1	Cocaine	↓ SIRT1 binding to numerous promoters		24 h	Chronic	Cocaine (20 mg/kg, i.p.) for 7 days	Ferguson et al., 2015	25698746
SIRT1	Cocaine	↑ mRNA	qPCR	24 h	Chronic	Cocaine (20 mg/kg, i.p.) for 7 days	Renthal et al., 2009	19447090
SIRT1	Cocaine	↑ SIRT1 activity		24 h	Chronic	Cocaine (20 mg/kg, i.p.) for 7 days	Renthal et al., 2009	19447090
SIRT1	Cocaine	↑ mRNA	qPCR	4 h	Chronic	Cocaine (20 mg/kg, i.p.) for 7 days	Ferguson et al., 2013	24107942
SIRT1	Cocaine	↑ mRNA	qPCR	5 days	Chronic	Cocaine (20 mg/kg, i.p.) for 7 days	Ferguson et al., 2013	24107942
SIRT1	Morphine	↑ mRNA	qPCR	24 h	Chronic	Morphine (20 mg/kg, i.p.) for 7 days	Ferguson et al., 2013	24107942
SIRT1	Morphine	↑ Protein	WB	24 h	Chronic	Morphine (20 mg/kg, i.p.) for 7 days	Ferguson et al., 2013	24107942
SIRT1	Morphine	↑ mRNA	qPCR	5 days	Chronic	Morphine (20 mg/kg, i.p.) for 7 days	Ferguson et al., 2013	24107942
SIRT2	Cocaine	↑ SIRT2 activity		24 h	Chronic	Cocaine (20 mg/kg, i.p.) for 7 days	Renthal et al., 2009	19447090
SIRT2	Cocaine	↑ mRNA	qPCR	24 h	Chronic	Cocaine (20 mg/kg, i.p.) for 7 days	Ferguson et al., 2013	24107942
SIRT2	Cocaine	↑ Protein	WB	24 h	Chronic	Cocaine (20 mg/kg, i.p.) for 7 days	Ferguson et al., 2013	24107942
SIRT2	Cocaine	↑ mRNA	qPCR	24 h	Chronic	Cocaine (20 mg/kg, i.p.) for 7 days	Renthal et al., 2009	19447090
SIRT2	Cocaine	↑ mRNA	qPCR	4 h	Chronic	Cocaine (20 mg/kg, i.p.) for 7 days	Ferguson et al., 2013	24107942
CBP	Cocaine	↑ CBP binding on cfos promoter	ChIP-qPCR	1 h	Acute	Cocaine (10 mg/kg, i.p.)	Malvaez et al., 2011	22114264
CBP	Cocaine	↑ CBP binding on cfos promoter	ChIP-qPCR	1 h	Chronic	Cocaine (10 mg/kg, i.p.), for 5 days	Malvaez et al., 2011	22114264
CBP	Ethanol	↓ mRNA	qPCR	18 h	Drinking	Chronic intermittent access two bottle choice 20% alcohol drinking 3 days per week for 4 weeks	Sharma et al., 2021	34837399
Myst3	Ethanol	↑ mRNA in high drinkers		6 h	Drinking	Ethanol drinking sessions (4 drinking and 4 days of abstinence, repeated four times)	Wolstenholme et al., 2011	21698166
Atf-2	Methamphe tamine	↑ protein	WB	1 h	Acute	METH (20 mg/kg, i.p.)	Martin et al., 2012	22470541
Atf-2	Methamphe tamine	↑ protein	WB	16 h	Acute	METH (20 mg/kg, i.p.)	Martin et al., 2012	22470541
Atf-2	Methamphe tamine	↑ protein	WB	2 h	Acute	METH (20 mg/kg, i.p.)	Martin et al., 2012	22470541
Atf-2	Methamphe tamine	↑ protein	WB	4 hrs	Acute	METH (20 mg/kg, i.p.)	Martin et al., 2012	22470541
Atf-2	Methamphe tamine	↑ protein	WB	8 hrs	Acute	METH (20 mg/kg, i.p.)	Martin et al., 2012	22470541
G9a/Ehmt2	Cocaine	↑ binding at Cdk5, p65/NFkB, FosB, promoters	ChIP-qPCR	1 hrs	Acute	Cocaine (20 mg/kg, i.p.)	Maze et al., 2010	20056891
G9a/Ehmt2	Cocaine	↓ binding at LIMK promoters	ChIP-qPCR	1 h	Acute	Cocaine (20 mg/kg, i.p.)	Maze et al., 2010	20056891
G9a/Ehmt2	Cocaine	↓ mRNA in Drd1	qPCR	2 h	Chronic	Cocaine (20 mg/kg, i.p.) for 8 days	Maze et al., 2014	24584053
G9a/Ehmt2	Cocaine	↓ mRNA in Drd2	qPCR	2 h	Chronic	Cocaine (20 mg/kg, i.p.) for 8 days	Maze et al., 2014	24584053
G9a/Ehmt2	Cocaine	↓ Protein	WB	24 h	Chronic	Cocaine (20 mg/kg, i.p.) for 7 days	Covington et al., 2011	21867882
G9a/Ehmt2	Cocaine	↓ mRNA	qPCR	24 h	Chronic	Cocaine (20 mg/kg, i.p.) for 7 days	Kennedy et al., 2013	23475113
G9a/Ehmt2	Cocaine	↓ binding at Cdk5, p65/NFkB, Arc, FosB, LIMK, BDNF, APRT promoters	ChIP-qPCR	24 h	Chronic	Cocaine (20 mg/kg, i.p.) for 7 days	Maze et al., 2010	20056891
G9a/Ehmt2	Cocaine	↓ mRNA	qPCR	24 h	Chronic	Cocaine (20 mg/kg, i.p.) for 7 days	Maze et al., 2010	20056891
G9a/Ehmt2	Cocaine, human	↓ protein	WB		Human	Human Post mortem	Maze et al., 2014	24584053
G9a/Ehmt2	Ethanol	↓ protein	WB	3 days	Chronic	Chronic intermittent alcohol vapor exposure	Anderson et al., 2021	34013595
G9a/Ehmt2	Ethanol	↓ mRNA in high drinkers		6 h	Drinking	Ethanol drinking sessions (4 drinking and 4 days of abstinence, repeated four times)	Wolstenholme et al., 2011	21698166
G9a/Ehmt2	Morphine	↓ mRNA	qPCR	24 h	Chronic	Morphine (20 mg/kg, i.p.) for 5 days	Sun et al., 2012;	23197736
G9a/Ehmt2	Morphine	↓ mRNA	qPCR	24 h	Chronic	Morphine (20 mg/kg, i.p.) for 7 days	Sun et al., 2012;	23197736
GLP/Ehmt1	Cocaine	↓ mRNA in Drd1	qPCR	2 h	Chronic	Cocaine (20 mg/kg, i.p.) for 8 days	Maze et al., 2014	24584053
GLP/Ehmt1	Cocaine	↓ mRNA	qPCR	24 h	Chronic	Cocaine (20 mg/kg, i.p.) for 7 days	Maze et al., 2010	20056891
Suv39h1 (KMT1A)	Amphetamine	↑ mRNA	qPCR	5 days	Chronic	Amphetamine (4 mg/kg, i.p.) for 7 days	Renthal et al., 2009	18632938
Mll1	Methamphe tamine	↑ mRNA	qPCR	1.5 h	Acute	METH with CPP conditining	Aguilar-Valles et al., 2014	24183790
Setd6	Ethanol	↓ mRNA	qPCR	0.5 h	Acute	Ethanol (2 g/kg, i.p), challenge at 17 days	Botia et al., 2012	23110077
Setd6	Ethanol	↓ mRNA, sensitized animals	qPCR	0.5 h	Chronic	Ethanol (2 g/kg, i.p), for 10 days + Ethanol challenge at 17 days	Botia et al., 2012	23110077
Smyd3	Ethanol	↓ mRNA	qPCR	0.5 h	Acute	Ethanol (2 g/kg, i.p), challenge at 17 days	Botia et al., 2012	23110077
Smyd3	Ethanol	↓ mRNA, sensitized animals	qPCR	0.5 h	Chronic	Ethanol (2 g/kg, i.p), for 10 days + Ethanol challenge at 17 days	Botia et al., 2012	23110077
PRMT1	Cocaine	↑ activity		0.5 h	Acute	Cocaine (20 mg/kg) with 3 CPP conditioning	Li Y. et al., 2015	26377474
PRMT1	Cocaine	↑ mRNA	qPCR	1 h	Acute	Cocaine (20 mg/kg, i.p.)	Li Y. et al., 2015	26377474
PRMT1	Cocaine	↓ mRNA	qPCR	24 h	Acute	Cocaine (20 mg/kg, i.p.)	Damez-Werno et al., 2016	27506785
PRMT1	Cocaine	↓ mRNA	qPCR	24 h	Chronic	Cocaine (20 mg/kg, i.p.) for 7 days	Damez-Werno et al., 2016	27506785
PRMT1	Cocaine	↑ mRNA	qPCR	24 h	Chronic	Cocaine (20 mg/kg, i.p.) for 7 days	Li Y. et al., 2015	26377474
PRMT1	Cocaine	↑ protein	WB	24 h	Chronic	Cocaine (20 mg/kg, i.p.) for 7 days	Li Y. et al., 2015	26377474
PRMT1	Cocaine	↑ mRNA	qPCR	24 h	SA	Cocaine SA	Li Y. et al., 2015	26377474
PRMT1	Cocaine	↑ protein	WB	24 h	SA	Cocaine SA	Li Y. et al., 2015	26377474
PRMT10	Ethanol	↑ mRNA	NanoString analysis	3 weeks	Chronic	Chronic intermittent alcohol vapor exposure	Johnstone et al., 2021	31373129
PRMT2	Cocaine	↓ mRNA	qPCR	24 h	Chronic	Cocaine (20 mg/kg, i.p.) for 7 days	Damez-Werno et al., 2016	27506785
PRMT4	Ethanol	↓ mRNA, withdrawal 3 weeks	NanoString analysis	3 weeks	Chronic	Chronic intermittent alcohol vapor exposure	Johnstone et al., 2021	31373129
PRMT5	Cocaine	↓ mRNA	qPCR	24 h	Chronic	Cocaine (20 mg/kg, i.p.) for 7 days	Damez-Werno et al., 2016	27506785
PRMT5	Ethanol	↓ mRNA	qPCR	0.5 h	Acute	Ethanol (2 g/kg, i.p), challenge at 17 days	Botia et al., 2012	23110077
PRMT5	Ethanol	↓ mRNA, sensitized animals	qPCR	0.5 h	Chronic	Ethanol (2 g/kg, i.p), for 10 days + Ethanol challenge at 17 days	Botia et al., 2012	23110077
PRMT6	Cocaine	↓ mRNA	qPCR	24 h	Chronic	Cocaine (20 mg/kg, i.p.) for 7 days	Damez-Werno et al., 2016	27506785
PRMT6	Cocaine	↓ mRNA	qPCR	24 h	Chronic	Cocaine (20 mg/kg, i.p.) for 7 days	Damez-Werno et al., 2016	27506785
PRMT6	Cocaine	↓ Protein	WB	24 h	Chronic	Cocaine (20 mg/kg, i.p.) for 7 days	Damez-Werno et al., 2016	27506785
PRMT6	Cocaine	↓ mRNA	qPCR	24 h	Chronic	Cocaine (20 mg/kg, i.p.) for 7 days	Li Y. et al., 2015	26377474
PRMT6	Cocaine	↓ mRNA	qPCR	28 days	Chronic	Cocaine (20 mg/kg, i.p.) for 7 days	Damez-Werno et al., 2016	27506785
PRMT6	Cocaine	↓ Protein	WB	7 days	SA	Cocaine SA	Damez-Werno et al., 2016	27506785
PRMT6	Cocaine, human	↓ mRNA	qPCR		Human	Human Post mortem	Damez-Werno et al., 2016	27506785
PRMT6	Ethanol	↓ mRNA	qPCR	0.5 h	Acute	Ethanol (2 g/kg, i.p), challenge at 17 days	Botia et al., 2012	23110077
PRMT6	Ethanol	↓ mRNA, sensitized animals	qPCR	0.5 h	Chronic	Ethanol (2 g/kg, i.p), for 10 days + Ethanol challenge at 17 days	Botia et al., 2012	23110077
PRMT7	Ethanol	↓ mRNA	qPCR	0.5 h	Acute	Ethanol (2 g/kg, i.p), challenge at 17 days	Botia et al., 2012	23110077
PRMT7	Ethanol	↓ mRNA, sensitized animals	qPCR	0.5 h	Chronic	Ethanol (2 g/kg, i.p), for 10 days + Ethanol challenge at 17 days	Botia et al., 2012	23110077
PRMT8	Cocaine	↓ mRNA	qPCR	24 h	Chronic	Cocaine (20 mg/kg, i.p.) for 7 days	Damez-Werno et al., 2016	27506785
PRMT9	Cocaine	↓ mRNA	qPCR	24 h	Chronic	Cocaine (20 mg/kg, i.p.) for 7 days	Damez-Werno et al., 2016	27506785
KDM6B	Ethanol	↑ protein	WB	3 weeks	Chronic	Chronic intermittent alcohol vapor exposure	Johnstone et al., 2021	31373129
KDM6B	Ethanol	↓ mRNA	qPCR	3 weeks	Chronic	Chronic intermittent alcohol vapor exposure	Johnstone et al., 2021	31373129
KDM6B	Ethanol, human	↑ mRNA - humans with AUD	qPCR		Human		Johnstone et al., 2021	31373129
								

### Effects of Drugs on Epigenetic Regulators in the Nucleus Accumbens in Humans

Notably, though we mainly only review rodent studies, several important pieces of evidence in post-mortem human NAc studies demonstrate that drug exposure induces alteration of many epigenetic regulators for histone acetylation and histone methylation. HDAC5 mRNA is downregulated in the NAc of people that use heroin (Egervari et al., 2017). In addition, the methyltransferases G9a and PRMT6 are downregulated in post-mortem NAc tissue from people that take cocaine (Maze et al., 2014; Damez-Werno et al., 2016). Finally, the histone lysine-specific demethylase KDM6B is upregulated in people diagnosed with alcohol use disorder (Johnstone et al., 2021). This down-regulation of methyltransferases and upregulation of demethylases may produce some similar changes in histone marks in the NAc of humans with SUD as compared to rodent models of SUD (Tables 1, 2), but these have not been closely examined yet.

### Effects of Addictive Drugs on Epigenetic Regulators in the Nucleus Accumbens in Rodent Models

As shown in Table 3 there are many known candidates that are regulated by drug exposure in the NAc.

#### Histone Deacetylase Proteins

Many histone deacetylase proteins (HDACs), including Class I (HDAC1, 2, 3, and 8), Class IIa (HDAC4, 5, 7, and 9), Class IIb (HDAC6 and 10), Class III (SirtI and II), and Class IV (HDAC11), are regulated by drug exposure as detailed below.

##### Class I Histone Deacetylase Proteins (HDAC1, 2, 3, and 8)

HDAC1 protein expression decreases after acute non-contingent methamphetamine exposure for at least 1-16 h (Martin et al., 2012). In contrast, chronic non-contingent cocaine exposure increases the enrichment of HDAC1 on G9a and GLP promoters at 4 hrs after the last drug exposure (Kennedy et al., 2013). Chronic non-contingent amphetamine exposure for consecutive 7 days increases HDAC1 enrichment on the cfos promoter at 5 days after the last exposure (Renthal et al., 2008). HDAC2 expression increases after acute non-contingent exposure to nicotine and methamphetamine, chronic non-contingent exposure to ethanol, and contingent self-administered cocaine (Host et al., 2011; Martin et al., 2012; Faillace et al., 2015; Torres et al., 2015; Sharma et al., 2021). HDAC3 expression and binding to some promoter regions decreases after acute non-contingent exposure to cocaine and methamphetamine (Rogge et al., 2013; Torres et al., 2016). Like HDAC2, chronic exposure to non-contingent cocaine increases HDAC3 expression and binding to some promoters (Campbell et al., 2021). Finally, HDAC8 expression decreases after acute non-contingent methamphetamine exposure (Torres et al., 2016). These data demonstrate that there are distinct effects of different drugs on the expression of class I HDACs, and that both contingent and non-contingent administration can alter HDACs.

##### Class IIa Histone Deacetylase Proteins (HDAC4, 5, 7, and 9)

Class IIa HDACs are also regulated by drug exposure. HDAC4 expression decreases after acute non-contingent methamphetamine exposure (Torres et al., 2016). Contingent ethanol drinking in rodents increases HDAC4 mRNA expression and decreases protein expression (Griffin et al., 2017; Pozhidayeva et al., 2020). HDAC5 decreases after non-contingent cocaine conditioned place preference conditioning (Rogge et al., 2013) and after contingent chronic ethanol exposure (Pozhidayeva et al., 2020). As noted above, HDAC5 mRNA is similarly downregulated in the NAc of people that use heroin (Egervari et al., 2017). HDAC7 expression decreases for at least 1-8 h following an acute methamphetamine exposure, like HDAC4 (Torres et al., 2016). HDAC9 expression decreases in rodents subjected to chronic non-contingent ethanol exposure after a withdrawal of 3 weeks (Johnstone et al., 2021).

##### Class IIb Histone Deacetylase Proteins (HDAC6 and 10)

HDAC6 mRNA increases after acute, non-contingent methamphetamine from 1-8 h after the exposure (Torres et al., 2016).

##### Class III (SirtI and II)

Increased Sirt1 expression and activity is observed at both 4-24 h and 5 days after chronic non-contingent cocaine exposure. Similarly, Sirt2 expression and activity increases after chronic non-contingent cocaine exposure (Renthal et al., 2009; Ferguson et al., 2015). Also, ChIP-seq with Sirt1 analysis identified changes in Sirt1 enrichment on some promoter regions after chronic non-contingent cocaine exposure (Ferguson et al., 2015).

##### Class IV Histone Deacetylase Proteins

HDAC11 mRNA expression changes after exposure to contingent cocaine self-administration (Host et al., 2011), non-contingent methamphetamine (Torres et al., 2016), and non-contingent ethanol exposure (Botia et al., 2012). In these studies, both acute non-contingent ethanol and chronic ethanol exposure decreases HDAC11 mRNA. Similarly, acute methamphetamine decreases mRNA expression from 1 to 8 hrs. In contrast, contingent cocaine self-administration increases HDAC11 expression at 2 hrs after the last drug exposure.

#### Histone Acetyltransferases

Histone acetyltransferases (HATs) are also regulated by drug exposure. Both acute and chronic non-contingent cocaine exposure increases the enrichment of Creb-binding protein (CBP) on a specific promoter (Malvaez et al., 2011) (see Table 3 for details). Also, chronic contingent ethanol exposure decreases CBP mRNA expression (Sharma et al., 2021). Finally, lysine acetyltransferase 6A, KAT6A (also known as Myst3) mRNA increases after contingent chronic exposure to ethanol (Wolstenholme et al., 2011) and Atf-2 increases following a non-contingent methamphetamine exposure (Martin et al., 2012).

#### Histone Lysine Methyltransferases

The histone methyltransferase G9a regulates several histone marks including H3K9me2 and G9a expression in the NAc is reduced by chronic exposure to non-contingent cocaine (Maze et al., 2014), non-contingent morphine (Sun et al., 2012), and both contingent/non-contingent alcohol models (Wolstenholme et al., 2011; Anderson et al., 2021). Consistent with decreasing G9a protein expression after chronic cocaine exposure, G9a enrichment on several gene promoters increase at 1 h after acute non-contingent cocaine exposure and decrease at 24 h after chronic cocaine exposure (Maze et al., 2010). As mentioned above, G9a is also downregulated in humans that use cocaine (Maze et al., 2014). Of note, another histone methyltransferase called G9a-like protein (GLP, also called EHMT1) also decreases following non-contingent cocaine exposure (Maze et al., 2010, 2014). Also, the lysine methyltransferase (KMT) KMT1A (also known as Suv39h1) increases after 7 days of non-contingent amphetamine exposure (Renthal et al., 2008). In addition, KMT2a (also known as Mll1) increases after non-contingent methamphetamine conditioned place preference conditioning (Aguilar-Valles et al., 2014), and the KMTs Setd6 and Smyd3 decrease following both acute and chronic exposure to non-contingent ethanol (Botia et al., 2012).

#### Protein Arginine Methyltransferases

Protein arginine methyltransferases (PRMTs) like PRMT1 to PRMT6 and PRMT8 to PRMT10 are also altered after drug exposure. PRMT1 was initially reported to increase expression and activity after acute and chronic non-contingent exposure to cocaine, and following contingent cocaine self-administration (Li Y. et al., 2015). In contrast, a later paper reported that PRMT1 decreases after acute and chronic non-contingent cocaine exposure (Damez-Werno et al., 2016), so there is some disagreement in this area. PRMT2, PRMT5, PRMT6, PRMT8, and PRMT9 decrease following chronic non-contingent cocaine exposure (Li Y. et al., 2015; Damez-Werno et al., 2016). PRMT6 also decreases 7 days after the last contingent cocaine self-administration and 28 days after the last non-contingent cocaine exposure (Damez-Werno et al., 2016), suggesting this may be a long-lasting change in the NAc. As noted above, PRMT6 mRNA is similarly downregulated in post-mortem samples from people that take cocaine. PRMT5, PRMT6, and PRMT7 mRNA expression decreases 30 min after a non-contingent ethanol challenge at 17 days after 10 days of chronic non-contingent ethanol exposure (Botia et al., 2012). Finally, PRMT4 decreases at least 3 weeks after chronic intermittent alcohol vapor exposure (Johnstone et al., 2021).

#### Other Epigenetic Regulators

In addition, other epigenetic regulators are also altered by drugs exposure like the lysine demethylase (KDM) KDM6B. KDM6B mRNA and protein expression were increase and decrease, respectively, at 3 weeks after chronic exposure to non-contingent ethanol (Johnstone et al., 2021). As noted above, KDM6B is similarly upregulated in humans with AUD.

#### Activity, Localization, Phosphorylation, and Binding Changes of Epigenetic Regulators

Most of the previously mentioned studies measure RNA or protein levels, however some have shown that drug exposure can alter other aspects of protein regulation like nuclear versus cytoplasmic localization of HDAC4 and HDAC5 (Renthal et al., 2009; Taniguchi et al., 2012; Penrod et al., 2018), phosphorylation (Renthal et al., 2009; Taniguchi et al., 2012; Penrod et al., 2018), or their binding activity to genes (Renthal et al., 2009; Maze et al., 2010; Levine et al., 2011; Malvaez et al., 2011; Kennedy et al., 2013; Rogge et al., 2013; Ferguson et al., 2015; Li Y. et al., 2015; Torres et al., 2015; Campbell et al., 2021). These changes suggest that simply examining the up- or -down regulation of mRNA and/or protein levels may be insufficient to understand how epigenetic regulators are altered by addictive drugs.

#### Conclusion

Many epigenetic regulator proteins are altered by drug exposure. These changes have been observed in rodent studies and human post-mortem studies as well. These findings suggest that at least some preclinical findings translate to the clinic. Finally, this suggests that treatments that can alter drug-related behaviors in preclinical studies may be beneficial clinically to treat SUD.

## Effects of Systemically Injected Inhibitors of Epigenetic Regulators on Drug-Related Behaviors

Many studies have altered drug-related behaviors in preclinical rodent studies by systemic or i.c.v. administration of inhibitors/activators of epigenetic proteins. As shown in Table 4, these studies have produced mixed results. The same or similar inhibitor compounds sometimes increase or decrease drug-related behaviors depending on the drug used or the behavioral procedure. We have organized Table 4 based on the type of inhibitor used and described the epigenetic target, drugs, behavioral model, and whether it was reported to increase or decrease drug-taking or drug-seeking behavior.

**TABLE 4:** Effects of systemic inhibitors of epigenetic regulators on drug-related behaviors.

Epigenetic Target	Drug	Manipulation	Behavior	Behavioral Effect	References	PMID
HDACs	Heroin	NaBut, non-specific inhbitor, i.c.v.	SA	↑ Heroin SA primed-reinstatement, 12 hrs before Heroin prime	Chen et al., 2016	27742468
HDACs	Ethanol	NaBut, non-specific inhbitor, i.c.v.	SA	↓ Ethanol SA in only dependent rats	Simon-O’Brien et al., 2015	25041570
HDACs	Cocaine	NaBut, non-specific inhbitor, systemic	CPP	↑ Cocaine CPP	Itzhak et al., 2013	23567105
HDACs	Cocaine	NaBut, non-specific inhbitor, systemic	CPP	↑ Cocaine CPP extinction	Malvaez et al., 2010	19765687
HDACs	Morphine	NaBut, non-specific inhbitor, systemic	CPP	↑ Morphine CPP	Sanchis-Segura et al., 2009	19727068
HDACs	Cocaine	NaBut, non-specific inhbitor, systemic	CPP	↓ Cocaine CPP Extinction	Itzhak et al., 2013	23567105
HDACs	Cocaine	NaBut, non-specific inhbitor, systemic	CPP	↓ Cocaine CPP primed reinstatement	Malvaez et al., 2010	19765687
HDACs	Nicotine	NaBut, non-specific inhbitor, systemic	CPP	↓ Nicotine CPP	Pastor et al., 2011	21166804
HDACs	Amphetamine	NaBut, non-specific inhbitor, systemic	Locomotor	↑ Amphetamine locomotor sensitization	Kalda et al., 2007	17477979
HDACs	Cocaine	NaBut, non-specific inhbitor, systemic	Locomotor	↑ Cocaine locomotor activity and sensitization	Kumar et al., 2005	16242410
HDACs	Morphine	NaBut, non-specific inhbitor, systemic	Locomotor	↑ Morphine sensitization	Sanchis-Segura et al., 2009	19727068
HDACs	Ethanol	NaBut, non-specific inhbitor, systemic	Locomotor	↓ Ethanol locomotor sensitization	Legastelois et al., 2013	23488934
HDACs	Cocaine	NaBut, non-specific inhbitor, systemic	SA	↑ Cocaine SA	Sun et al., 2008	18599214
HDACs	Heroin	NaBut, non-specific inhbitor, systemic	SA	↑ Heroin SA primed-reinstatement, 12 hrs before Heroin prime	Chen et al., 2016	27742468
HDACs	Cocaine	NaBut, non-specific inhbitor, systemic	SA	↓ Cocaine SA reinstatement (cue + cocaine combination)	Romieu et al., 2011	21886555
HDACs	Ethanol	NaBut, non-specific inhbitor, systemic	SA	↓ Ethanol SA in only dependent rats	Simon-O’Brien et al., 2015	25041570
HDACs	Ethanol	TSA, non-specific inhbitor, systemic	Anxiety	↓ Ethanol withdrawal-induced anxiety	Pandey et al., 2008	18385331
HDACs	Ethanol	TSA, non-specific inhbitor, systemic	Drinking	↑ Two-bottle Ethanol intake	Wolstenholme et al., 2011	21698166
HDACs	Ethanol	TSA, non-specific inhbitor, systemic	Drinking	↓ Ethanol consumption	Sakharkar et al., 2014	24528596
HDACs	Ethanol	TSA, non-specific inhbitor, systemic	Drinking	↓ Ethanol intake (drinking in the dark)	Warnault et al., 2013	23423140
HDACs	Cocaine	TSA, non-specific inhbitor, systemic	CPP	↑ Cocaine CPP	Kumar et al., 2005	16242410
HDACs	Cocaine	TSA, non-specific inhbitor, systemic	Locomotor	↓ Cocaine locomotor sensitization	Romieu et al., 2008	18799668
HDACs	Amphetamine	TSA, non-specific inhbitor, systemic	SA	↓ Amphetamine SA cue-induced reinstatement in socially isolated rats	Arndt et al., 2019	31343201
HDACs	Amphetamine	TSA, non-specific inhbitor, systemic	SA	↓ Amphetamine SA cue-reinstatement in socialy isolated rats	Arndt et al., 2019	31343201
HDACs	Cocaine	TSA, non-specific inhbitor, systemic	SA	↓ Cocaine SA intake	Host et al., 2010	20132486
HDACs	Cocaine	TSA, non-specific inhbitor, systemic	SA	↓ Cocaine SA intake and motivation	Romieu et al., 2008	18799668
HDACs	Cocaine	Phenylbutyrate, non-specific inhbitor, systemic	SA	↓ Cocaine SA intake	Romieu et al., 2008	18799668
HDACs	Cocaine	Depudecin, non-specific inhbitor, systemic	SA	↓ Cocaine SA motivation	Romieu et al., 2008	18799668
HDACs (class I and II)	Ethanol	VPA, selective inhibitor, systemic	Drinking	↓ Ethanol consumption and preference in 2-bottle choice	Al Ameri et al., 2014	25108044
HDACs (class I and II)	Ethanol	VPA, selective inhibitor, systemic	CPP	↓ Ethanol CPP	Al Ameri et al., 2014	25108044
HDACs (class I and II)	Amphetamine	VPA, selective inhibitor, systemic	Locomotor	↑ Amphetamine locomotor sensitization	Kalda et al., 2007	17477979
HDACs (class I and II)	Ethanol	SAHA, selective inhibitor, systemic	Drinking	↓ Ethanol intake (drinking in the dark), but not saccharin	Warnault et al., 2013	23423140
HDACs (class I and II)	Cocaine	SAHA, selective inhibitor, systemic	CPP	↑ Cocaine CPP	Renthal et al., 2007	17988634
HDACs (class I and II)	Morphine	SAHA, selective inhibitor, systemic	CPP	↑ Morphine CPP extinction	Saberian et al., 2021	34302880
HDACs (class I and II)	Morphine	SAHA, selective inhibitor, systemic	CPP	↓ Morphine primed-reinstatement in CPP	Saberian et al., 2021	34302880
HDACs (class I and II)	Ethanol	SAHA, selective inhibitor, systemic	SA/Drinking	↓ Ethanol SA presses and intake, but not sucrose	Warnault et al., 2013	23423140
HDACs (class I and II)	Ethanol	SAHA, selective inhibitor, systemic	SA/Drinking	↓ Ethanol drug-seeking during EXT training, but not sucrose	Warnault et al., 2013	23423140
HDACs (class I)	Ethanol	MS275, selective inhibitior, i.c.v.	Drinking	↓ Ethanol consumption, lever presses, motivation, ↓ relapse	Jeanblanc et al., 2015	25762717
HDACs (class I)	Ethanol	MS275, selective inhibitior, systemic	Drinking	↓ Ethanol intake (drinking in the dark)	Warnault et al., 2013	23423140
HDACs (class II)	Cocaine	MC1568, inhibitor, systemic	SA	↑ Cocaine SA motivation and punishment resistance	Griffin et al., 2017	29109977
HDAC1 and HDAC2	Amphetamine	Cpd-60, selective inhibitior, systemic	Locomotor	↓ Amphetamine locomotion	Schroeder et al., 2013	23967191
HDAC3	Cocaine	RGFP966, selective inhibitor, systemic	CPP	↑ Cocaine CPP extinction	Malvaez et al., 2013	23297220
HDAC3	Cocaine	RGFP966, selective inhibitor, systemic	CPP	↓ Cocaine CPP reinstatement	Malvaez et al., 2013	23297220
HDAC3	Cocaine	RGFP966, selective inhibitor, systemic	SA	↓ Cocaine SA reinstatement	Hitchcock et al., 2019	30488346
HDAC4/5	Ethanol	LMK235, HDAC4/5 inhibitor, systemic	Drinking	↓ Ethanol binge-like drinking	Pozhidayeva et al., 2020	32085427
HDACs	Morphine	Theophylline, selective activator, systemic	CPP	↓ Morphine CPP extinction	Saberian et al., 2021	34302880
SIRTs	Cocaine	Resveratrol, agoinst, systemic	CPP	↑ Cocaine CPP	Renthal et al., 2009	19447090
G9a	Ethanol	UNC0642, selective inhibitor, systemic	Drinking	↓ stress-escalated Ethanol drinking	Anderson et al., 2021	34013595

### Histone Deacetylase Protein Activators/inhibitors

Many different compounds that alter HDAC activity have been injected systemically to study their effects on drug-related behaviors.

#### Histone Deacetylase Protein Inhibitor: Sodium Butyrate

The non-selective HDAC inhibitor sodium butyrate (NaBut) has been shown to alter many drug-related behaviors, but can produce mixed results. For instance, NaBut increases non-contingent psychostimulant- and morphine-induced locomotor sensitization, but in contrast decreases ethanol-induced locomotor sensitization (Kumar et al., 2005; Kalda et al., 2007; Sanchis-Segura et al., 2009; Legastelois et al., 2013). In addition, NaBut increases cocaine and morphine conditioned place preference (CPP) (Sanchis-Segura et al., 2009; Itzhak et al., 2013) and decreases cocaine CPP extinction (Itzhak et al., 2013) in some papers. However, there is some disagreement as other studies show that NaBut increases cocaine CPP extinction (Malvaez et al., 2010), reduces primed reinstatement in cocaine CPP (Malvaez et al., 2010), and decreased nicotine CPP (Pastor et al., 2011). The effects of NaBut administration are thus inconsistent between these studies on non-contingent drug-related behaviors.

The effects of NaBut on contingent self-administration behaviors are also inconsistent as some papers report that NaBut increases cocaine self-administration and increases heroin prime-induced reinstatement (Sun et al., 2008; Chen et al., 2016), but others report it decreases reinstatement to cocaine-seeking and decreases alcohol drinking (Romieu et al., 2011; Simon-O’Brien et al., 2015). These discrepancies could be due to the non-selective nature of NaBut or differences in experimental design.

#### Histone Deacetylase Protein Inhibitor: Trichostatin A

The non-selective HDAC inhibitor trichostatin A (TSA) also alters drug-related behaviors, but not all studies are consistent. TSA increases cocaine CPP (Kumar et al., 2005) and also increases contingent ethanol intake (Wolstenholme et al., 2011), however, other reports suggest that TSA reduces ethanol drinking (Sakharkar et al., 2014), reduces ethanol withdrawal-induced anxiety (Pandey et al., 2008), and reduces psychostimulant self-administration, sensitization, and reinstatement (Romieu et al., 2008; Host et al., 2010; Arndt et al., 2019). Again, these discrepancies could be due to the non-selective nature of TSA or differences in experimental design.

#### Other Histone Deacetylase Protein Inhibitors

Less well studied non-selective HDAC inhibitors like phenylbutyrate and depudecin have been shown to reduce contingent cocaine self-administration (Romieu et al., 2008).

#### Selective Class I and II Histone Deacetylase Protein Inhibitors

Given the many differences in studies following the use of non-selective HDAC inhibitors, more selective inhibitors that act only on a subset of HDACs have also been studied (Table 4). As described above, HDACs can be divided into several classes and Class I includes HDAC1, HDAC2, HDAC3, and HDAC8. Class II includes HDAC4-HDAC7 and HDAC9-HDAC10. Both valproic acid (VPA) and suberoylanilide hydroxamic acid (SAHA) are selective for only these classes of HDACs and not other class III and IV HDACs like SIRT1-7 and HDAC11. These two inhibitors more consistently reduce drug-related behaviors. SAHA reduces contingent ethanol intake, ethanol self-administration, ethanol-seeking (Warnault et al., 2013), and SAHA also increases extinction and reduces non-contingent CPP reinstatement to morphine (Saberian et al., 2021). Like SAHA, VPA also reduces ethanol drinking and ethanol CPP (Al Ameri et al., 2014). However, other studies report that SAHA increases cocaine CPP (Renthal et al., 2007) and VPT increases amphetamine locomotor sensitization (Kalda et al., 2007). These divergent effects could be due to these drugs acting on many different targets.

#### Class Selective Histone Deacetylase Protein Inhibitors

Inhibitors selective for Class I HDACs (MS275) or Class II HDACs (MC1568) have also been used to alter drug-related behaviors. Interestingly, the Class 1 inhibitor reduces ethanol drinking (Warnault et al., 2013), ethanol self-administration, and reinstatement to ethanol-seeking (Jeanblanc et al., 2015), whereas the Class II inhibitor increases the motivation for cocaine self-administration (Griffin et al., 2017) suggesting different roles for these HDAC classes.

#### More Selective HDAC Inhibitors

Moving from classes to specific proteins, Compound 60 is a selective inhibitor of HDAC1 and HDAC2 and reduces acute non-contingent amphetamine locomotor behavior (Schroeder et al., 2013). Also, RGFP966 is a selective inhibitor of HDAC3 and increases CPP extinction, blocks reinstatement (Malvaez et al., 2013), and also reduces reinstatement to cocaine seeking (Hitchcock et al., 2019). Finally, LMK235 is an HDAC4/HDAC5 inhibitor that reduces contingent ethanol intake (Pozhidayeva et al., 2020). In general, these reports suggest that more selective HDAC inhibitors may be more consistent in reducing cocaine-seeking and ethanol intake behaviors, though they are still understudied at this point.

#### Histone Deacetylase Protein Activators

Activators of HDACs have also been studied, but rarely. The HDAC activator theophylline decreases extinction to non-contingent morphine CPP and (Saberian et al., 2021) the SIRT agonist resveratrol increases non-contingent cocaine CPP (Renthal et al., 2009). Since these studies suggest that HDAC activation increases drug-related behaviors, they complement some of the HDAC inhibitor studies that show decreases in drug-related behaviors.

#### Histone Deacetylase Protein Inhibitor/Activator Conclusions

Examining all these HDAC inhibitor/activator studies together, it is not possible to draw a strong conclusion on their effects on behavior. This could be due to these systemically administered compounds affecting many different brain and/or body regions, but could also be due to differences in experimental design and timing of exposure.

### G9a Inhibitors

The systemic G9a inhibitor UNC0642 has recently been shown to reduce stress-induced alcohol drinking (Anderson et al., 2021), suggesting other epigenetic regulators can be targeted with systemic injections as well.

## Effects of Nucleus Accumbens-Specific Epigenetic Regulator Manipulations on Drug-Related Behaviors

Since systemic inhibitors likely alter many brain regions, NAc-specific manipulations are more helpful to determine the specific effect of epigenetic regulator proteins in this brain region. Many epigenetic modifiers have been targeted in a NAc-specific manner as thoroughly described in Table 5.

**TABLE 5:** Effects of NAc-specific epigenetic regulator manipulations on drug-related behaviors.

Epigenetic Target	Drug	Manipulation	Behavior	Behavioral Effect	References	PMID
HDACs	Cocaine	TSA, non-specific inhbitor, intra-Nac	SA	↑ Cocaine SA motivation	Wang et al., 2010	20010550
HDACs	Cocaine	TSA, non-specific inhbitor, intra-Nac	SA	↑ Cocaine SA sensitivity	Wang et al., 2010	20010550
HDACs	Ethanol	TSA, non-specific inhbitor, intra-Nac	Locomotor	↑ Ethanol lomotor behavior	Sprow et al., 2014	25130590
HDACs	Heroin	TSA, non-specific inhbitor, intra-Nac	CPP	↑ Heroin CPP	Sheng et al., 2011	21734607
HDACs	Cocaine	TSA, non-specific inhbitor, intra-Nac	SA	↓ Cocaine SA reinstatement (cue + cocaine combination)	Romieu et al., 2011	21886555
HDACs	Ethanol	TSA, non-specific inhbitor, intra-Nac	Drinking	↓ Ethanol intake (drinking in the dark)	Warnault et al., 2013	23423140
HDACs	Amphetamine	VPA, inhibitor, intra-Nac	Locomotor	↓ amphetamine locomotor sensitization	Kim et al., 2008	18164815
HDACs (class I and II)	Cocaine	SAHA, selective inhibitor, intra-Nac	CPP	↑ Cocaine CPP	Renthal et al., 2007	17988634
HDACs (class I and II)	Cocaine	SAHA, selective inhibitor, intra-Nac	SA	↑ Cocaine SA motivation	Wang et al., 2010	20010550
HDACs (class I and II)	Cocaine	SAHA, selective inhibitor, intra-Nac	SA	↑ Cocaine SA sensitivity	Wang et al., 2010	20010550
HDACs (class I)	Cocaine	MS275, selective inhibitior, intra-Nac	Locomotor	↓ Cocaine locomotor sensitization	Kennedy et al., 2013	23475113
HDAC1	Cocaine	Floxed HDAC1 mice, intra-Nac Cre	Locomotor	↓ Cocaine locomotor sensitization	Kennedy et al., 2013	23475113
HDAC3	Cocaine	Floxed HDAC3 mice, intra-Nac AAV-cre	CPP	↑ Cocaine CPP acquisition	Rogge et al., 2013	23575859
HDAC3	Cocaine	HDAC3 Y298H overexpression in D1	CPP	↑ Cocaine CPP	Campbell et al., 2021	33602824
HDAC3	Cocaine	HDAC3 Y298H overexpression in D1	SA	↓ Cocaine seeking withdrawal Day1 and Day 30	Campbell et al., 2021	33602824
HDAC4	Cocaine	Cytoplasmic HDAC4 overexpression	CPP	↑ Cocaine CPP	Penrod et al., 2018	28635037
HDAC4	Cocaine	Floxed HDAC4 mice, intra-Nac AAV-Cre	Locomotor	↓ Acute cocaine locomotor activity	Penrod et al., 2018	28635037
HDAC4	Cocaine	Floxed HDAC4 mice, intra-Nac AAV-Cre	CPP	↓ Cocaine CPP	Penrod et al., 2018	28635037
HDAC4	Cocaine	Floxed HDAC4 mice, intra-Nac AAV-Cre	Locomotor	↓ Cocaine locomotor sensitization	Penrod et al., 2018	28635037
HDAC4	Cocaine	HDAC4 overexpression	CPP	↓ Cocaine CPP	Kumar et al., 2005	16242410
HDAC4	Cocaine	HDAC4 overexpression	SA	↓ Cocaine SA motivation	Wang et al., 2010	20010550
HDAC4	Cocaine	HDAC4 overexpression	SA	↓ Cocaine SA sensitivity	Wang et al., 2010	20010550
HDAC5	Cocaine	HDAC5 constitutive KO mouse	CPP	↑ Cocaine CPP	Renthal et al., 2007	17988634
HDAC5	Cocaine	HDAC5 overexpression	CPP	↓ Cocaine CPP	Renthal et al., 2007	17988634
HDAC5	Cocaine	Nuclear HDAC5 overexpression	CPP	↓ Cocaine CPP	Taniguchi et al., 2012	22243750
HDAC5	Cocaine	Nuclear HDAC5 overexpression	CPP	↓ Cocaine CPP	Taniguchi et al., 2017	28957664
HDAC5	Cocaine	Nuclear HDAC5 overexpression	SA	↓ Cocaine SA cue and primed RN	Taniguchi et al., 2017	28957664
SIRT1	Cocaine	Floxed SIRT1 mice, intra-Nac AAV-Cre	CPP	↓ Cocaine CPP	Ferguson et al., 2013	24107942
SIRT1	Morphine	Floxed SIRT1 mice, intra-Nac AAV-Cre	CPP	↓ Morphine CPP	Ferguson et al., 2013	24107942
SIRT1	Cocaine	SIRT1 Overexpression	CPP	↑ Cocaine CPP	Ferguson et al., 2013	24107942
SIRT1	Cocaine	SIRT1 Overexpression	CPP	↑ Morphine CPP	Ferguson et al., 2013	24107942
SIRT1	Cocaine	SIRT1 Overexpression	Locomotor	↑ Cocaine locomoter behavior	Ferguson et al., 2013	24107942
SIRT2	Cocaine	SIRT2 Overexpression	CPP	↑ Cocaine CPP	Ferguson et al., 2013	24107942
SIRT2	Cocaine	SIRT2 Overexpression	CPP	↑ Morphine CPP	Ferguson et al., 2013	24107942
SIRTs	Cocaine	Sirtinol, antagonist, intra-Nac	CPP	↓ Cocaine CPP	Renthal et al., 2009	19447090
SIRTs	Cocaine	Sirtinol, antagonist, intra-Nac	SA	↓ Cocaine SA, reduces dose response	Renthal et al., 2009	19447090
CBP	Cocaine	Floxed CBP mice, intra-Nac AAV-cre	Locomotor	↓ Acute cocaine locomotor activity	Malvaez et al., 2011	22114264
CBP	Cocaine	Floxed CBP mice, intra-Nac AAV-cre	CPP	↓ Cocaine CPP	Malvaez et al., 2011	22114264
CBP	Cocaine	Floxed CBP mice, intra-Nac AAV-cre	Locomotor	↓ Cocaine locomotor sensitization	Malvaez et al., 2011	22114264
G9a/Ehmt2	Cocaine	BIX01294, inhibitor, intra-Nac	CPP	↑ Cocaine CPP	Maze et al., 2010	20056891
G9a/Ehmt2	Cocaine	Floxed G9a mice, intra-Nac Cre	CPP	↑ Cocaine CPP	Maze et al., 2010	20056891
G9a/Ehmt2	Morphine	Floxed G9a mice, intra-Nac Cre	Locomotor	↑ Morphine locomotor sensitization	Sun et al., 2012	23197736
G9a/Ehmt2	Cocaine	G9a overexpression	SA	↑ Cocaine SA sensitivity, motivation	Anderson et al., 2018a	29217682
G9a/Ehmt2	Cocaine	G9a overexpression	SA	↑ stress-induced reinstatement	Anderson et al., 2018a	29217682
G9a/Ehmt2	Cocaine	G9a overexpression	CPP	↓ Cocaine CPP	Maze et al., 2010	20056891
G9a/Ehmt2	Morphine	G9a overexpression	Locomotor	↓ Morphine CPP and locomotor sensitization	Sun et al., 2012	23197736
G9a/Ehmt2	Cocaine	G9a overexpression only in D2-MSNs	CPP	↓ Cocaine CPP	Maze et al., 2014	24584053
G9a/Ehmt2	Cocaine	shRNA-mediated G9a knockdown	SA	↓ Cocaine drug-seeking (context-, drug primed-, and stress-induced)	Anderson et al., 2019	30587852
G9a/Ehmt2	Cocaine	shRNA-mediated G9a knockdown	SA	↓ Cocaine SA sensitivity, motivation	Anderson et al., 2019	30587852
G9a/Ehmt2	Ethanol	shRNA-mediated G9a knockdown	Drinking	↓ Ethanol drinking (stress-induced)	Anderson et al., 2021	34013595
PRMT1	Cocaine	AMI-1, selective inhibitor, intra-Nac	CPP	↓ Cocaine CPP	Li Y. et al., 2015	26377474
PRMT1	Cocaine	Knockdown with LV short hairpin	CPP	↓ Cocaine CPP	Li Y. et al., 2015	26377474
PRMT1	Cocaine	MTA, selective inhibitor, intra-Nac	CPP	↓ Cocaine CPP	Li Y. et al., 2015	26377474
PRMT1	Cocaine	SKLB-639, selective inhibitor, intra-Nac	CPP	↓ Cocaine CPP	Li Y. et al., 2015	26377474
PRMT6	Cocaine	miRNA knockdown in D2	CPP	↓ Cocaine CPP	Damez-Werno et al., 2016	27506785
PRMT6	Cocaine	PRMT6 overexpression in D2	CPP	↑ Cocaine CPP	Damez-Werno et al., 2016	27506785
PRMT6	Cocaine	PRMT6 overexpression in D1	CPP	↓ Cocaine CPP	Damez-Werno et al., 2016	27506785
KDM5C	Methamphetamine	siRNA-mediated KDM5C knockdown in Nac	CPP	↓ Methamphetamine CPP	Aguilar-Valles et al., 2014	24183790
Mll1	Methamphetamine	siRNA-mediated Mll1 knockdown in Nac	CPP	↓ Methamphetamine CPP	Aguilar-Valles et al., 2014	24183790

### Nucleus Accumbens-Specific Injections of Histone Deacetylase Protein Inhibitors

Several of the epigenetic inhibitors discussed above have also been injected into the NAc specifically including TSA, VPA, SAHA, and MS275. Some data suggest that injecting the non-selective HDAC inhibitors TSA and VPA into the NAc reduces drug-related behaviors like cocaine reinstatement (Romieu et al., 2011), ethanol drinking (Warnault et al., 2013), and amphetamine locomotor sensitization (Kim et al., 2008). In contrast, others suggest the opposite as TSA increases heroin CPP (Sheng et al., 2011), increases ethanol-induced locomotor behavior (Sprow et al., 2014), and increases the motivation for cocaine and cocaine sensitivity using self-administration assays (Wang et al., 2010).

The more selective HDAC inhibitors SAHA and MS275 have also been injected in the NAc. The Class I and Class II HDAC inhibitor SAHA increases CPP (Renthal et al., 2007) and increases the motivation for cocaine and cocaine sensitivity (using dose-response testing) as measured with contingent cocaine self-administration assays (Wang et al., 2010). Finally, the selective Class I HDAC inhibitor MS275 reduces locomotor sensitization when injected into the NAc (Kennedy et al., 2013). These studies demonstrate a NAc-specific effect of epigenetic regulation on drug-related behaviors, however, cannot determine which epigenetic proteins (or combination of proteins) are responsible for these effects.

### Nucleus Accumbens-Specific Overexpression and/or Knockdown of Histone Deacetylase Proteins

To understand the role of individual epigenetic regulators in the NAc, many investigators have site-specifically altered the expression of a target protein and examined its effects on drug-related behaviors.

#### HDAC4

The first evidence of a specific functional epigenetic protein acting in the NAc was that overexpressing HDAC4 decreases cocaine CPP (Kumar et al., 2005). Later studies suggested that overexpressing HDAC4 - but not a catalytic HDAC-domain deletion mutant - reduces the motivation for cocaine as well (Wang et al., 2010), suggesting HDAC4 reduces cocaine reward-seeking behaviors. However, there are still inconsistencies as in contrast, other studies in *Hdac4* NAc conditional knockout mice show HDAC4 increases drug-related behaviors like CPP and sensitization (Penrod et al., 2018).

#### HDAC5

Other class IIb HDAC studies show that HDAC5 blocks cocaine CPP (Renthal et al., 2007; Taniguchi et al., 2012) and reduces the reinstatement of drug-seeking behavior following cocaine self-administration (Taniguchi et al., 2017). As described above, cocaine exposure induces the dephosphorylation and nuclear accumulation of HDAC5 in a cAMP-dependent manner in D1-containing medium spiny neurons (D1-MSNs). The dephosphorylated nuclear-accumulated form of HDAC5, but not wild-type HDAC5, limits drug-related behaviors. These data suggest a MSN-cell-type dependent HDAC5 function on drug-related behaviors and in response to drug exposure.

#### HDAC3

The Class I HDAC, HDAC3 also alters drug-related behavior. A NAc-specific conditional knockout of HDAC3 in mice increases cocaine CPP (Rogge et al., 2013). A follow up study demonstrated a D1-MSN cell-type-specific role of HDAC3 in cocaine CPP as a mutated deacetylase activity-dead HDAC3 overexpressed only in NAc increases cocaine CPP. In the same study, the mutated deacetylase activity-dead HDAC3 also attenuates the reinstatement of drug-seeking behavior following cocaine self-administration (Campbell et al., 2021) suggesting discrepancies in the effects of epigenetic regulators on contingent vs non-contingent drug-related behaviors depending on the model used.

#### SIRTs

Class III HDACs (SIRTs) also regulate drug-related behavior as SIRT1 or SIRT2 overexpression increases cocaine and morphine CPP (Ferguson et al., 2013). Also, a *Sirt1* conditional knockout in mice reduces cocaine and morphine CPP (Ferguson et al., 2013). Finally, a NAc-specific injection of the SIRT antagonist sirtinol reduces cocaine CPP and cocaine self-administration (Renthal et al., 2009).

### Nucleus Accumbens-Specific Overexpression and/or Knockdown of HATs

Histone acetyltransferases in the NAc also function in cocaine-related behaviors as a NAc conditional knockout of CBP in mice decreases cocaine locomotor activity and cocaine CPP (Malvaez et al., 2011).

### Nucleus Accumbens-Specific Overexpression and/or Knockdown of Histone Methyltransferases

Moving from acetylation to methylation, initial studies suggested that the methyltransferase G9a reduces drug-induced locomotor sensitization and drug-conditioned place preference since overexpressing G9a blocks cocaine CPP (Maze et al., 2010, 2014), morphine CPP, and morphine locomotor sensitization (Sun et al., 2012). In addition, intra-NAc administration of the G9a inhibitor BIX01294 and *G9a* NAc conditional knockout increases cocaine CPP (Maze et al., 2010). Also, a NAc conditional knockout of *G9a* increases morphine CPP and morphine locomotor sensitization (Sun et al., 2012). However, later studies using contingent cocaine self-administration as a model demonstrated that overexpressing G9a in the NAc increases cocaine sensitivity (using dose-response testing), motivation (using progressive ratio testing), and stress-induced reinstatement (Anderson et al., 2018a). A subsequent study showed that reducing NAc G9a levels via shRNA reduces the sensitivity to cocaine self-administration, motivation, and stress-induced reinstatement (Anderson et al., 2019). Together, these studies showed that G9a levels in the NAc have bi-directional effects on cocaine self-administration and cocaine-seeking behaviors (Anderson et al., 2018a,^2019^). In addition, reducing G9a in the NAc also blocks stress-induced ethanol drinking and this effect is recapitulated by systemic administration of UNC0642 - a selective G9a inhibitor – as mentioned above (Anderson et al., 2021). These studies again suggest that at least some differences in preclinical studies could be explained by differences in contingent vs non-contingent models.

### Other Epigenetic Modifiers

Other methyltransferases like PRMT1 alter cocaine CPP as shown by PRMT1 knockdown and pharmacological inhibition studies (Li Y. et al., 2015). Also, PRMT6 overexpression in the NAc increases cocaine CPP, and miRNA-mediated knockdown reduces cocaine CPP (Damez-Werno et al., 2016). Finally, another study shows that KDM5C or Mll1 knockdown reduces methamphetamine CPP (Aguilar-Valles et al., 2014).

### Conclusion

These studies and others in Table 5 demonstrate the powerful effects that epigenetic regulators can have on drug-related behaviors in pre-clinical models. However, these reports often conflict concerning the function of these NAc-specific manipulations, suggesting that differences in experimental design (like the use of contingent vs non-contingent models) can produce different behavioral effects. Still, these reports suggest that translating some of these methods into the clinic could potentially help to reduce the negative effects of substance use.

## Limitations and Challenges in the Field of Epigenetics and Substance Use Disorder

### Diversity of Epigenetic Modifications and Their Substrates

Despite the large amount of data on epigenetic regulation in rodent models of SUD, and the ability to alter drug-related behaviors through either systemically administered or NAc-specific manipulations, there are still many limitations and challenges for the field. Beginning with examining changes in drug-induced epigenetic modifications, we think that there is a great need for more unbiased approaches. For instance, after initial studies found changes in H3 and H4 PTMs, many subsequent studies only examined these sites with specific antibodies. While these studies often found changes, the focus on these known sites could have prevented the discovery of other important sites of regulation, especially considering the wide array of time-dependent and substance-dependent changes shown in Tables 1, 2. These biases extended to a concentrated study of just a handful of histone PTMs when there are over 100 histone PTMs, most of which have not been examined following exposure to addictive drugs. For example, there are reports on other epigenetic markers like histone phosphorylation that can be altered following cocaine (Bertran-Gonzalez et al., 2008), morphine (Ciccarelli et al., 2013), and methamphetamine use (Rotllant and Armario, 2012). In addition, poly-ADP-ribosylation of histones is altered by drug exposure (Scobie et al., 2014). Notably, two novel histone modifications, serotonylation (Farrelly et al., 2019) and dopaminylation of H3Q5 (Lepack et al., 2020), have been recently reported and could play a role in NAc-mediated drug-related behaviors. Dopaminylation of H3Q5 in the VTA is dysregulated by cocaine exposure and may alter cocaine self-administration behavior (Lepack et al., 2020) and could play a role in the NAc as well. Given the recent reports of these novel histone PTMs, it is possible that we are still missing other important PTMs too.

Some of these issues are due to technical challenges like the need for better antibodies to other PTMs. ChIP assays are limited by the available antibodies so even large “unbiased” approaches have an inherent bias based on these tools (Renthal et al., 2009; Feng et al., 2014) and truly unbiased genome wide PTMs analyses are not yet possible. In addition to examining the epigenetic modifications that influence the transcriptome, chromatin accessibility could be examined by unbiased genome-wide approaches using an Assay for Transposase-Accessible Chromatin (ATAC)-seq or DNase-seq (Fullard et al., 2018; Carullo et al., 2020; Scherma et al., 2020). These assays could be coupled with other unbiased approaches to examine the downstream effects of these targets on transcriptional changes like RNA-seq and/or microarrays to get closer to a complete picture of the effects of these epigenetic changes produced by drugs exposure. Indeed, many of the studies examined in this review did not report on transcriptomic changes and only examined histone PTMs or select gene expression changes. Finally, very little is known of the actual protein changes (and not just mRNA changes) that occur following these epigenetic manipulations and future studies should examine the functional outcomes of these epigenetic effects.

Other forms of epigenetic regulation not involving direct histone PTM regulation can also influence drug-related behaviors. Though out of the scope of this review, DNA methylation is another form of epigenetic regulation that can be altered by drug exposure for weeks after the last drug exposure. In addition, manipulating proteins associated with DNA methylation can also alter drug-related behaviors (see (Werner et al., 2021) and (Anderson et al., 2018b) for reviews). Also, other types of cellular regulation, including non-coding RNAs (ncRNA), are beginning to be understood extensively (Gu et al., 2021). For example, long ncRNAs can be regulated by cocaine at least 24 hrs after the last injection (Bu et al., 2012). Also, microRNAs are regulated by cocaine, heroin, and methamphetamine in the NAc (Eipper-Mains et al., 2011; Su et al., 2019; Dash et al., 2020; Yang et al., 2020; Li et al., 2021; Xu et al., 2021) and the dorsal striatum (Hollander et al., 2010; Im et al., 2010). Finally, small nucleolar RNAs (snoRNAs) are also regulated by cocaine and knockdown of MBII-52 attenuates cocaine CPP (Chen et al., 2014). Taking a broader look at epigenetic regulation of these RNA subtypes could help us determine which epigenetic mechanisms should be targeted to reduce the negative effects of SUD in humans.

### Cell Type Specific Epigenetic Regulations

Another major limitation of almost all studies to date is that they do not separate different cellular populations like neurons vs glia. Drug-induced molecular and synaptic plasticity alterations occur in specific cell types to drive behavioral changes (Lobo et al., 2010; Pascoli et al., 2011; Maze et al., 2014; Campbell et al., 2021), but these are often overlooked in whole NAc tissue punches. Not only are glia often included in these epigenetic assays, but neurons that may not be involved in drug-related behaviors are also included. Only small populations of neurons that have been activated during drug-related learning, called engrams, seem to be important for various drug-related behaviors (Koya et al., 2009; Cruz et al., 2014; Hsiang et al., 2014). In contrast, most epigenetic and molecular studies examine whole tissue in the NAc following drug exposure. This means that all cells are being studied in these analyses including dopamine receptor D1 or D2-containing medium spiny neurons (D1- or D2-MSNs), interneurons, glial cells, microglia, and even some amount of blood vessel and blood cells. This hodgepodge of cells may be limiting our ability to detect the specific changes relating to SUD. Perhaps the subset of important cells that drive addictive behavior do retain a distinct histone methylation or acetylation signature, but this is diluted by other cells that return to baseline thus limiting our ability to detect lasting changes that are still present.

Reducing this signal to noise ratio is possible thanks to technologies like cre-driven gene expression or single cell RNA-seq (Macaulay et al., 2017). Several studies have examined differences in D1- and D2-MSN cell type specific regulation of epigenetic mechanisms. These studies suggest distinct epigenetic regulation in specific cell types. For instance, HDAC3, G9a, and PRMT6 have unique roles in D1 vs D2-MSN cell type specific manner (Maze et al., 2014; Damez-Werno et al., 2016; Campbell et al., 2021). Also, cocaine increased H3 phosphorylation only in D1 cells according to one report (Bertran-Gonzalez et al., 2008). Using a combination of cell-type specific transcriptomic analyses and cre-dependent cell lines will be able to further elucidate the role of epigenetic regulation in D1- and D2-MSNs in SUD. Technologies like Fos-Targeted Recombination in Active Population (TRAP) and ArcTRAP allow for examining groups of cells that are regulated together through activity. This technique was recently used and found that despite no overall changes in dorsal striatum HDAC4 and HDAC5 mRNA levels, these transcripts were altered in FOS-positive neurons following prolonged methamphetamine self-administration withdrawal (Li X. et al., 2015). These current advanced technologies enable us to examine transcription and open chromatin status at the single-cell or single-nucleus level. Combining these techniques with RNA-seq, ATAC-seq, or ChIP-seq could also provide data on epigenetic changes at the single-cell level (Rotem et al., 2015). These powerful techniques could help to determine more specific roles of epigenetic regulation caused by exposure to addictive drugs.

### Effects of Contingent and Non-contingent Drugs Exposure

Some differences discussed in this review may be due to issues in the rodent models used in these studies. As mentioned above, rodent models can be broadly separated into two classes: (1) experimenter administered (non-contingent) models, including CPP, locomotor sensitization, and alcohol vapor exposure where the rodents have no choice in drug exposure or (2) self-administration (contingent) models that allow the rodents more choice over when to take drugs. These assays include alcohol drinking (2-bottle choice, drinking-in-the-dark) and drug self-administration. Sometimes, these contingent and non-contingent experimental models indicate a similar role of epigenetic regulators in the development of drug reward-conditioned behaviors. For example, HDAC5 has similar roles on drug-related behavior following both non-contingent cocaine CPP and contingent reinstatement of cocaine-seeking behaviors after cocaine SA (Taniguchi et al., 2017). In addition, HDAC4 overexpression in the NAc decreases cocaine intake and reduces motivation in the progressive ratio schedule in a contingent self-administration model (Wang et al., 2010) and also decreases non-contingent cocaine CPP (Kumar et al., 2005). In contrast, in a later study, Hdac4 NAc conditional knockout mice exhibited decreases in cocaine-induced locomotor activity, sensitization, and cocaine CPP in non-contingent experiments (Penrod et al., 2018). Disagreements in the literature like these examples are often found and can lead to very different interpretations. For example, G9a NAc conditional knockout mice exhibited increases in cocaine CPP and overexpressing G9a decreases cocaine CPP suggesting G9a reduces the cocaine-induced behavioral plasticity using non-contingent models (Maze et al., 2010). However, in a contingent cocaine self-administration model, G9a overexpression increases sensitivity in dose-response test, motivation in progressive ratio testing, and stress-induced reinstatement, suggesting that G9a increases cocaine-related behaviors (Anderson et al., 2018a). Since behavioral differences are observed using similar manipulations of epigenetic regulators, it is important to examine the effects of epigenetic manipulations in a variety of behavioral tasks to find those that may be more likely to translate to humans.

## Future Directions and Conclusion

As shown above, we now know drug exposure regulates histone marks and epigenetic regulators. Most of these changes appear to be very short-lived, but some can be long-lasting (at least a month) especially when examining changes at specific promoters (Damez-Werno et al., 2012, 2016; Tomasiewicz et al., 2012; Flagel et al., 2016; Carpenter et al., 2020; Johnstone et al., 2021). However, we still do not understand the mechanisms that promote some changes and not others, and it is often difficult to tell if these changes are functional and whether they are addiction-promoting or counter adaptive protective mechanisms (Anderson et al., 2018a). Fortunately, new technologies are being developed that allow for the selective targeting of specific genomic loci (Heller et al., 2014, 2016; Hamilton et al., 2018), these cutting-edge tools allow for epigenetic regulation of a single gene and allow very precise control of gene expression in neurons. Targeting epigenetic mechanisms, possibly through systemic administration of protein inhibitors (Anderson et al., 2021), viral vectors like AAV in select groups of neurons like cell-type or engram-specific circuits - using the methods described in Guenthner et al. (2013), Maze et al. (2014), Damez-Werno et al. (2016), Campbell et al. (2021) - could lead to breakthrough future translational therapeutics in SUD.

## Author Contributions

EA and MT wrote sections of the manuscript and organized the database. Both authors contributed to manuscript revision, read, and approved the submitted version.

## Conflict of Interest

EA is a co-founder of NeuroEpigenix, LLC. The author declares that the research was conducted in the absence of any commercial or financial relationships that could be construed as a potential conflict of interest.

## Publisher’s Note

All claims expressed in this article are solely those of the authors and do not necessarily represent those of their affiliated organizations, or those of the publisher, the editors and the reviewers. Any product that may be evaluated in this article, or claim that may be made by its manufacturer, is not guaranteed or endorsed by the publisher.
